# Comprehensive Red List Assessment Reveals Exceptionally High Extinction Risk to Madagascar Palms

**DOI:** 10.1371/journal.pone.0103684

**Published:** 2014-07-30

**Authors:** Mijoro Rakotoarinivo, John Dransfield, Steven P. Bachman, Justin Moat, William J. Baker

**Affiliations:** 1 Kew Madagascar Conservation Centre, Ambodivoanjo Ivandry, Antananarivo, Madagascar; 2 Royal Botanic Gardens, Kew, Richmond, Surrey, United Kingdom; Institute of Agronomy, University of Lisbon, Portugal

## Abstract

The establishment of baseline IUCN Red List assessments for plants is a crucial step in conservation planning. Nowhere is this more important than in biodiversity hotspots that are subject to significant anthropogenic pressures, such as Madagascar. Here, all Madagascar palm species are assessed using the IUCN Red List categories and criteria, version 3.1. Our results indicate that 83% of the 192 endemic species are threatened, nearly four times the proportion estimated for plants globally and exceeding estimates for all other comprehensively evaluated plant groups in Madagascar. Compared with a previous assessment in 1995, the number of Endangered and Critically Endangered species has substantially increased, due to the discovery of 28 new species since 1995, most of which are highly threatened. The conservation status of most species included in both the 1995 and the current assessments has not changed. Where change occurred, more species have moved to lower threat categories than to higher categories, because of improved knowledge of species and their distributions, rather than a decrease in extinction risk. However, some cases of genuine deterioration in conservation status were also identified. Palms in Madagascar are primarily threatened by habitat loss due to agriculture and biological resource use through direct exploitation or collateral damage. The recent extension of Madagascar’s protected area network is highly beneficial for palms, substantially increasing the number of threatened species populations included within reserves. Notably, three of the eight most important protected areas for palms are newly designated. However, 28 threatened and data deficient species are not protected by the expanded network, including some Critically Endangered species. Moreover, many species occurring in protected areas are still threatened, indicating that threatening processes persist even in reserves. Definitive implementation of the new protected areas combined with local community engagement are essential for the survival of Madagascar’s palms.

## Introduction

Madagascar is one of the World’s most threatened biodiversity hotspots [1] because of the high endemism of its biota coupled with widespread habitat degradation, especially in humid forest areas. Despite ongoing scientific studies that have highlighted Madagascar as a place of endemic megadiversity that is facing intensifying extinction risk [2], the island’s charismatic flora and fauna remain under immense pressure [3], [4]. Conservation baselines are urgently required to demonstrate and strengthen the case for action on the ground.

Palms are among the most conspicuous components of the flora of Madagascar. To date, 195 species in 17 genera are recognized [5], [6] with all but three being endemic to the island (98% endemism). The palm flora of Madagascar is outstandingly rich in a global context [7]. Palms inhabit mostly primary vegetation although a few species occur in disturbed areas, such as anthropogenic grassland. Consistent with global patterns of palm distribution, 90% of Madagascar palms are restricted to humid forest [8], [9].

Palms are particularly vulnerable to humid forest degradation. In most species, survival and recruitment are reduced when habitat quality declines [10], [11] or when habitats become fragmented [12]. The extensive degradation of Madagascar’s humid forests, which have been reduced to around 25% of their original extent [13], implies that the island’s humid forest-restricted biota, such as palms, are likely to be extremely threatened.

In addition to habitat loss, palms are further threatened by unsustainable, targeted exploitation by humans. Alongside grasses and legumes, palms are among the most important plant families for humans [14], providing numerous useful resources, such as materials for construction or weaving, food, medicine and ornamental plants [15]. Palms play a particularly important role in poorer countries, such as Madagascar, where they have immense economic importance at the village level [10], but they are often destructively harvested, e.g. for palm heart consumption or construction materials. In recent decades, Madagascar palms have also been targeted by plant collectors for introduction to horticultural trade [16]. These human activities place palms at greater risk of extinction than other humid forest groups that are not exploited in this way.

Extinctions at both species and population levels are of concern because unique evolutionary history and ecosystem services may be lost [17], which is particularly significant in the case of keystone groups such as palms [9]. To prevent such biodiversity loss in Madagascar palms, a critical conservation strategy is required to focus attention on conservation priorities, to stimulate necessary actions and to raise public awareness. To take these steps, species of concern must first be properly identified based on sound taxonomy [18] so that accurate and cost-effective conservation management decisions can be made. The conservation performance of protected area networks can be improved with such information. Much of Madagascar’s biodiversity is unlikely to survive unless it occurs within protected areas [19], [20]. In 2003, the Madagascar government decided to increase the protected areas surface [21] from 1.7 million hectares (3%) [22] to 6 million hectare (10% of the island’s surface [23]) as many unprotected areas were found to be critically important for biodiversity [24], [25].

The International Union for the Conservation of Nature (IUCN) curates the IUCN Red List of Threatened Species, which is the most comprehensive, objective and authoritative data source on extinction risk in species [26]–[28]. Through the application of a set of five criteria (e.g. restricted range, declining population), a species can be classified according to its relative risk of extinction. In an earlier assessment of the palms of Madagascar [10], in which previous versions of the IUCN system [29], [30] were applied, 113 species were identified as threatened and 18 presumed extinct. In this paper, we present a complete and updated conservation assessment of all palm species in Madagascar using the current IUCN Red List categories and criteria (version 3.1 [31]). This work builds upon a robust taxonomy for the group established in recent years [10], [32], [33], [34] and a comprehensive database of collections and observations from recent field work [6].

The objective of this study is to produce a baseline conservation dataset for palms in Madagascar including taxonomy, species distributions, ecological factors and economic uses. We analyze this dataset to answer the following questions: 1) what is the current extinction risk to Madagascar’s endemic palm species, 2) how does current status compare with the previous assessment in 1995, 3) is the existing protected area network effective for palms and 4) what are the major threats to palms?

## Methods

### Study area

**Figure 1:**
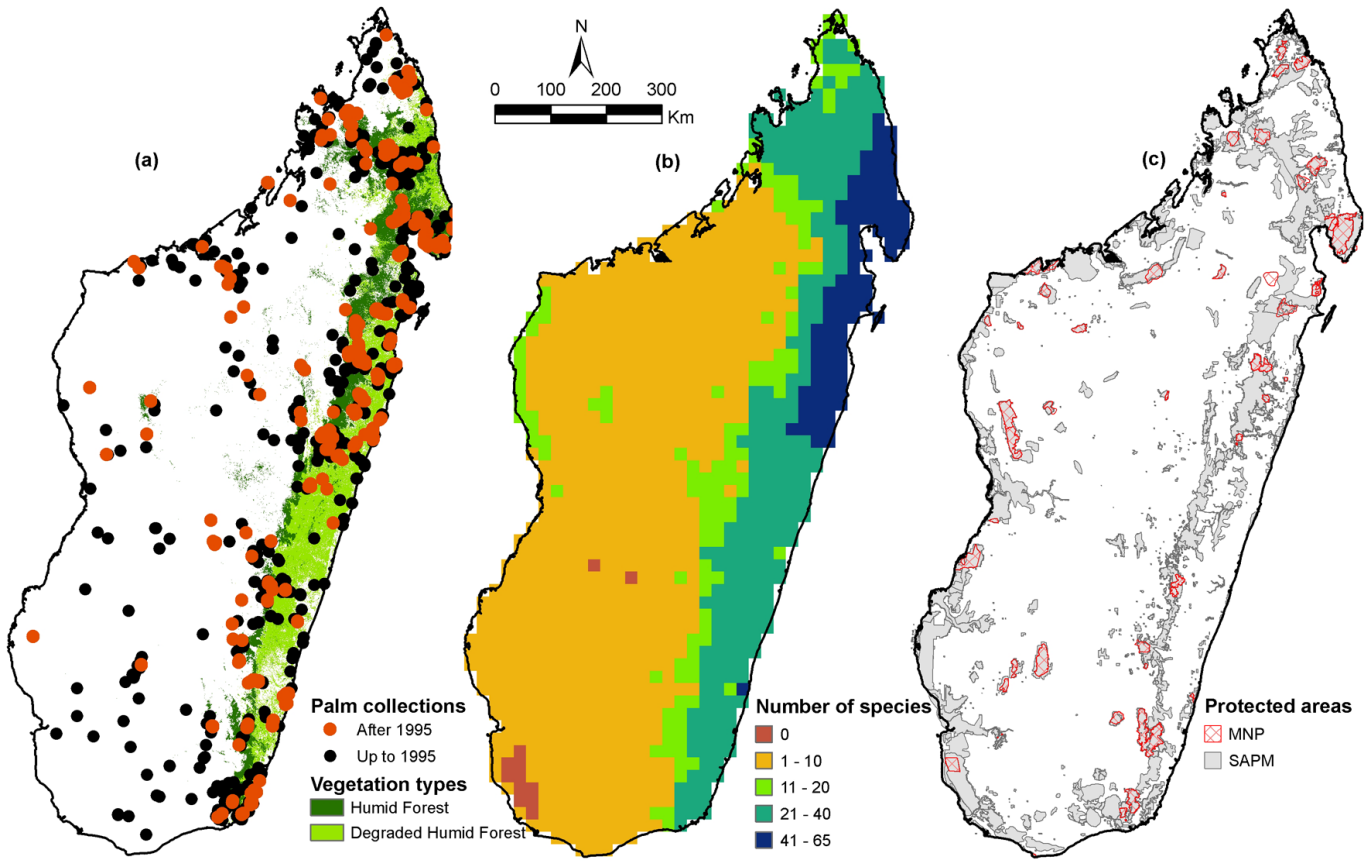
Palm distributions, humid forest and protected areas in Madagascar. (a) Palm specimen collection localities in Madagascar and extent of humid forest vegetation [13]. (b) Species richness of palms in Madagascar [6] illustrating predicted number of palm species across the island at a resolution of 0.2° (ca. 22 km × 22 km). (c) Protected area network in Madagascar comprising the long-standing MNP network (46 parks and reserves [50]) and the newly established SAPM (145 reserves, including those of the MNP network [23]).

Madagascar is a large tropical island (592,750 km^2^) in the Indian Ocean [35] and is the third largest tropical island in the world, after New Guinea and Borneo. The island has a complex landscape [36] and is dominated by mountains running north-south, resulting in a central highland region above 800 m elevation. On the eastern side of the central highland is an escarpment that falls steeply away towards the Indian Ocean, whereas the western side consists of a large plain declining gently to the Mozambique Channel. Due to the impact of the southeastern tradewinds (Alizé) and the northwestern monsoon from the Equator, the eastern region is humid to perhumid, the highlands are relatively temperate, the western region is subhumid to dry, and the far south-west is subarid [37]. Consequently, the island has a great diversity of primary vegetation types, ranging from humid forest to dry spiny forest (Fig. 1a) [13]. Humid forest, the primary habitat of most palms, is restricted to the east and north-west of the island. Of the estimated 21 million inhabitants, nearly 80% live in rural areas [38] and depend on natural resources for their subsistence, contributing to the destruction of Madagascar’s forests, which have declined by 40% between 1950 and 2000 alone [39].

### Occurrence data

The study is based on a dataset of 2,160 georeferenced occurrence records, derived from herbarium specimens of Madagascar palms in key botanical institutions around the world: AAU, FTG, GE, K, MO, NY, P, TAN, TEF and ZT (herbarium acronyms follow [40]). Collection dates range from 1834 to 2010. Records lacking geographic coordinates on specimen labels were georeferenced using topographic maps, online gazetteers, the Madagascar gazetteer of the Missouri Botanical Garden [41] and online mapping tools such as Google Earth [42].

Of the georeferenced records, 820 (38%) postdate 1995 when the previous conservation assessment was conducted [10] of which 561 (26%) result from our own fieldwork in Madagascar since 1995 (fig. 1a). Building on the robust taxonomic baseline provided by Dransfield and Beentje [10], we have conducted targeted fieldwork in 32 sites (Table 1) across Madagascar between 1995–2010 (Fig. 1a), focusing mostly on primary forest areas far from high human density where the palm flora is rich, but poorly known (Fig. 1b). This fieldwork has substantially improved our understanding of the distribution and the populations of 152 species (78% of the total palm flora). The number of specimen records per species ranges from 1 to 85 (mean: 10 specimens per species) and 107 species are known from fewer than 5 specimen records.

**Table 1:** Fieldwork locations visited by the authors.

Location	Latitude and longitudeco-ordinates of sites visited	Status
Ambakireny	17.69° S 48.01° E	Local community forest
Ambatovaky	16.86° S 49.26° E	Special Reserve
Ambodivoahangy (Makira)	15.28° S 49.62° E	Local community forest
Analalava (Mahajanga)	14.76 S° 47.43° E	Local community forest
Andilamena	16.98° S 48.84° E; 16.81° S 48.68° E	Local community forest
Anosibe an’Ala	19.66° S 48.11° E	Local community forest
Betafo	20.20° S 46.50° E	Local community forest
Betampona	17.91° S 49.20° E	Special Reserve
Brickaville	18.89° S 49.12° E; 18.96° S 48.85° E	Local community forest
Daraina	13.26° S 49.59° E	SAPM Reserve (managed by Fanamby)
Fenoarivo Atsinanana	17.29° S 49.41° E	SAPM Reserve (managed by Ecole Supérieure des Sciences Agronomiques- Forêts, Antananarivo)
Fort-Dauphin	24.77° S 47.18° E	Private Land (managed by Qit Minerals Madagascar/Rio Tinto)
	24.56° S 47.20° E	SAPM Reserve (managed by Asity Madagascar/Bird Life International)
Analalava (Foulpointe)	17.71° S 49.45° E	SAPM Reserve (managed by Missouri Botanical Gardens)
Ifanadiana	21.33° S 47.71° E	Local community forest
Itremo	20.57° S 46.56° E	SAPM Reserve (managed by Kew Madagascar Conservation Centre)
Maevatanana	16.76° S 47.03° E	Local community forest
Makira	15.38° S 49.44° E; 15.28° S 49.44° E	SAPM Reserve (managed by Wildlife Conservation Society)
Manakara	21.83° S 47.90° E	Local community forest
Mananara Avaratra	15.94° S 49.54° E	Local community forest
Mangerivola	18.20° S 48.92° E	Special Reserve
Mantadia	18.88° S 48.44° E	National Park
Masoala	15.31° S 49.85° E; 15.73° S 49.96° E,15.74° S 50.19° E, 15.77° S 50.07° E	National Park
Midongy Atsimo	23.55° S 47.08° E	National park
Soanierana Ivongo	16.68° S 49.60° E	Local community forest
Vondrozo	22.80° S 47.18° E	Local community forest

All fieldwork was conducted with prior informed consent of the necessary authorities (Table 1). Permission for all fieldwork activities was obtained from the Ministry of Environment and Forests (Ministère de l’Environnement et des Forêts). Additional permissions were required depending on the status of the area visited. For National Parks and Special Reserves, additional permits were issued by Madagascar National Parks (MNP). For Système des Aires Protégées de Madagascar (SAPM) Reserves, additional fieldwork permission was sought from the specific management authority of each site (Table 1). For Local Community Forests, the local village council (Communauté de Base, COBA) was consulted on arrival. Fieldwork on private lands required permission from the land owners in advance (Table 1). Herbarium specimens from our fieldwork were deposited at the Madagascar national herbarium at Parc Botanique et Zoologique de Tsimbazaza (TAN) and the Royal Botanic Gardens, Kew (K). Additional duplicates, where available, were distributed primarily to the Missouri Botanical Garden (MO) and the Natural History Museum, Paris (P).

### IUCN Red List Conservation Assessments

We conducted a complete assessment of the conservation status of all 192 endemic Madagascar palm species using the IUCN Red List categories and criteria, version 3.1 [31] with reference to the latest guidelines [43]. Assessments were independently reviewed and verified by the IUCN Palm Specialist Group Red List Authority and IUCN Red List Unit. They were subsequently published on-line on the IUCN Red List on 17 October 2012 [44]. All assessments are now accessible via the IUCN Red List web portal at www.iucnredlist.org.

Each species was classified according to one of the following IUCN categories: Extinct in the Wild (EW), Critically Endangered (CR), Endangered (EN), Vulnerable (VU), Near Threatened (NT), Least Concern (LC) or Data Deficient (DD). We used data from our palm occurrence dataset to summarise distribution, population size and threats to species in order to apply the quantitative Red List criteria. Although attempts were made to apply all five criteria in the Red List system (A, declining population; B, geographic range size and fragmentation, decline, or fluctuations; C, small population size and fragmentation, decline or fluctuations; D, very small population; E, quantitative analysis of extinction risk), as recommended by IUCN, most assessments were conducted using criterion B due to the limitations of available data, a common pattern for Red List assessments of plants and some other groups [45].

The palm occurrences were carefully scrutinised for georeference precision, taxonomic identification and likelihood of a population still being extant e.g. historical collections in areas now deforested were excluded. The geographic range of each species was then quantified using two metrics, extent of occurrence (EOO) and area of occupancy (AOO) [31], both of which can be used for assessments under criterion B (restricted range species). EOO was calculated by constructing the minimum convex polygon (convex hull) around known occurrences [46], [47] using the Conservation Assessment Tools extension to ArcView [48]. AOO was calculated with the same tools by overlaying a grid and interpreting known occurrences as occupied grid cells. The sum of occupied grid cells equates to the AOO value. A grid cell size of 2 × 2 km^2^ was applied, as recommended by IUCN [43], where sampling effort was deemed sufficient. In some cases, larger cell sizes were used (up to 10 × 10 km^2^ [25], [46]) to account for inadequate sampling across the range. These larger grid cells were not scaled down to the reference scale of 2 × 2 km^2^, so the assessments assume the distribution is fully saturated at the 2 × 2 km^2^ reference scale [43]. In cases where a species was known from less than three unique collection sites, EOO could not be calculated and AOO alone was estimated. For species known to occur at a single locality and in a well defined habitat, AOO was estimated by considering the available suitable habitat. Satellite imagery from Google Earth [42] was used to determine suitable areas and polygons were drawn to estimate area of occupancy (AOO).

To infer population trends, such as continuing decline or fragmentation of distribution range through time, GIS layers of the vegetation maps of Humbert & Cours-Darne [49] and Moat & Smith [13] were compared. The rate of the decline of the population of each species in the 42 years between these two baseline vegetation surveys (1965 and 2007) was then calculated from the loss of suitable habitat under its EOO and AOO.

In order to evaluate the trend in conservation status change over time, we compared the 2012 assessment [44] with the previous assessment made by Dransfield & Beentje [10]. The two assessments were based on different versions of the IUCN Red List categories and criteria. The 1995 assessment was broadly based on version 2.3 [29], whereas the 2012 assessment used version 3.1 [31]. While most Red List categories were comparable between the two assessments, the category “Rare” used by Dransfield and Beentje [10] comes from a scheme pre-dating version 2.3 and could not be related to a category in version 3.1. The category “Near Threatened” of version 3.1 was absent from version 2.3. “Not Threatened” (NotT), as used in the 1995 assessment, was regarded as equivalent to “Least Concern” in version 3.1. The change in the IUCN assessments was quantified for data sufficient species (i.e. those that had enough data to carry out a full assessment) that were assessed in both years and in comparable categories. Changes were sorted into three classes: a) no change, if the category of the species was the same in the two assessments, b) downlisted, if the assessment of extinction risk decreased, i.e. from higher to lower category (e.g. EN to VU) and c) uplisted, if the assessment of extinction risk increased, i.e. from lower to higher category (e.g. VU to EN).

### Protected area coverage

We compared the distribution of all species to the protected area network in order to assess the effectiveness and coverage of reserves for palm conservation. We used GIS layers (Fig. 1c) describing the 46 established protected areas within the MNP network [50] and the new protected area network being established by SAPM since 2011, which comprises 145 reserves, including those of MNP [23]. We assessed the relative threat status of species occurring in zero, one and two or more protected area to test the expectation that species occurring in fewer protected areas have higher threat ratings.

### Threats

During the assessment process the dominant threats for each species were classified according to the IUCN Threats Classification Scheme (version 3.2) [51]. Details about the threats and the local utilization of each species were obtained from expert field observations, specimen labels and literature sources. These data were later compiled to evaluate the relative importance of major threatening processes affecting palms in Madagascar.

## Results

### IUCN Red List Conservation Assessments

**Figure 2:**
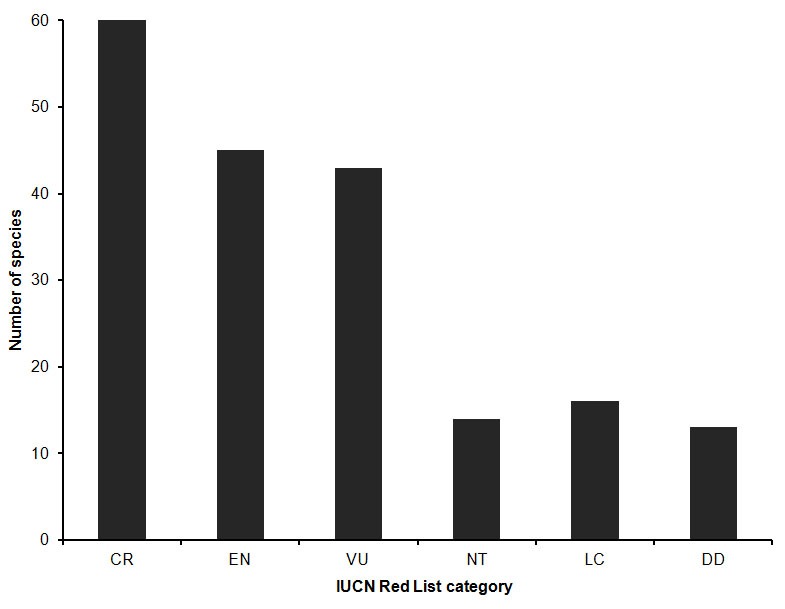
Summary of the 2012 IUCN Red List Assessments of Madagascar Palms (see **table 2**). IUCN Red List categories: Extinct in the Wild (EW), Critically Endangered (CR), Endangered (EN), Vulnerable (VU), Near Threatened (NT), Least Concern (LC), Data Deficient (DD) [31].

The results of our complete assessment of the conservation status of all known Madagascar palms are summarised in Table 2 and Figure 2, with a detailed break-down given in Table 3. Of the data sufficient species (179), we found that 149 (78%) are classified as threatened (CR, EN or VU). Thirteen species were not data sufficient and were thus rated as DD. Data on the current status of these species were inadequate to complete an assessment primarily because most were known only from the type collection and have not been observed for many years. Taking into account the 13 DD species, we estimated ‘lower’, ‘best estimate’ (‘mid-point’) and ‘upper’ bounds of the percentage of threatened species [52], which were 78%, 83% and 84% respectively. The lower bound treats all DD species as unthreatened, whereas the upper bound assumes that all are threatened. The best estimate assumes that the same fraction of DD species are threatened as was found for data sufficient species. A total of 14 species were listed as NT, which gives a total of 163 (91%) species considered to be of elevated conservation concern. Only 16 species were listed as LC.

**Table 2:** Summary of results from the 2012 IUCN Red List Assessment of Madagascar Palms.

	Count	Percentage
**IUCN Red List category**		
Extinct (EX)	0	0
Extinct in the Wild (EW)	0	0
Critically Endangered (CR)	61	32
Endangered (EN)	45	23
Vulnerable (VU)	43	22
Near Threatened (NT)	14	7
Least Concern (LC)	16	8
Data Deficient (DD)	13	7
**Summary Statistics**		
Total Evaluated	192	100
Total Data Sufficient (CR+EN+VU+NT+LC)	179	93
**Total Threatened** – lower bound (CR+EN+VU)	149	78
**Total Threatened** – best estimate (mid-point) (CR+EN+VU+((Total Threatened/Data Sufficient) × DD))	160	83
**Total Threatened** – upper bound (CR+EN+VU+DD)	162	84
Total Species of Elevated Conservation Concern (CR+EN+VU+NT)	163	85
Total Not Threatened (LC+DD)	30	16

**Table 3:** The conservation status of all 192 endemic Madagascar palm species.

Species	2012 Red List Assessment	1995 Red List Assessment	EOO (km^2^)	AOO (km^2^)	Status change	Populations in protected areas: MNP only (%)	Populations in protected areas: SAPM (%)
*Beccariophoenix alfredii*	VU	–	8	5	Described post-1995	0	0
Beccariophoenix madagascariensis	VU	CR	15460	300	Downlisted	30	80
*Bismarckia nobilis*	LC	NotT	319415	17100	No change	20	60
*Borassus madagascariensis*	EN	VU	48872	350	Uplisted	0	40
*Dypsis acaulis*	EN	EW	72	8	Downlisted	30	30
*Dypsis acuminum*	EN	DD	12113	150	Not comparable	50	80
*Dypsis albofarinosa*	CR	–	4	4	Described post-1995	100	100
*Dypsis ambanjae*	CR	EW	1767	150	Downlisted	50	100
*Dypsis ambilaensis*	EN	EN	176	60	No change	0	60
*Dypsis ambositrae*	CR	CR	1790	150	No change	0	30
*Dypsis andapae*	EN	Rare	1428	35	Not comparable	70	70
*Dypsis andilamenensis*	CR	–	10	5	Described post-1995	0	0
*Dypsis andrianatonga*	VU	Rare	5909	288	Not comparable	90	90
*Dypsis angusta*	EN	EN	5760	123	No change	40	70
*Dypsis angustifolia*	EN	Rare	469	327	Not comparable	30	100
*Dypsis anjae*	CR	–	4	4	Described post-1995	100	100
*Dypsis ankaizinensis*	DD	DD	–	–	No change	–	–
*Dypsis ankirindro*	NT	–	14	4	Described post-1995	0	100
*Dypsis antanambensis*	CR	EN	6	6	Uplisted	100	100
*Dypsis aquatilis*	CR	EN	35	25	Uplisted	0	0
*Dypsis arenarum*	CR	CR	895	36	No change	0	70
*Dypsis baronii*	LC	NotT	239065	6075	No change	40	70
*Dypsis basilonga*	CR	EN	188	16	Uplisted	0	0
*Dypsis beentjei*	CR	EN	6	6	Uplisted	100	100
*Dypsis bejofo*	VU	EN	10322	150	Downlisted	60	80
*Dypsis bernierana*	VU	VU	14610	600	No change	50	60
*Dypsis betamponensis*	VU	EW	4	4	Downlisted	100	100
*Dypsis betsimisarakae*	VU	–	7995	512	Described post-1995	50	80
*Dypsis boiviniana*	EN	EN	7196	896	No change	30	60
*Dypsis bonsai*	VU	VU	14420	520	No change	80	90
*Dypsis bosseri*	EN	EW	4	1.5	Downlisted	0	90
*Dypsis brevicaulis*	CR	CR	380	135	No change	0	80
*Dypsis brittiana*	CR	–	4	4	Described post-1995	0	100
*Dypsis canaliculata*	CR	EW	4114	243	Downlisted	30	70
*Dypsis canescens*	DD	EW	–	–	Not comparable	–	–
*Dypsis carlsmithii*	CR	–	2041	8	Described post-1995	50	100
*Dypsis catatiana*	LC	NotT	146761	3300	No change	70	80
*Dypsis caudata*	CR	CR	4	4	No change	100	100
*Dypsis ceracea*	EN	EW	11211	225	Downlisted	40	100
*Dypsis commersoniana*	DD	CR	–	–	Not comparable	–	–
*Dypsis concinna*	NT	VU	30591	2100	Downlisted	30	100
*Dypsis confusa*	NT	Rare	49262	1216	Not comparable	60	70
*Dypsis cookei*	CR	EN	6	4	Uplisted	100	100
*Dypsis coriacea*	NT	VU	11642	910	Downlisted	80	90
*Dypsis corniculata*	EN	VU	18147	1350	Uplisted	40	40
*Dypsis coursii*	LC	VU	3737	256	Downlisted	70	100
*Dypsis crinita*	NT	Rare	67442	1134	Not comparable	60	90
*Dypsis culminis*	EN	–	564	10	Described post-1995	0	100
*Dypsis curtisii*	EN	DD	7185	64	Not comparable	60	90
*Dypsis decaryi*	VU	VU	339	83	No change	40	40
*Dypsis decipiens*	VU	EN	42846	1430	Downlisted	20	40
*Dypsis delicatula*	VU	–	8	8	Described post-1995	40	80
*Dypsis digitata*	CR	CR	2334	8	No change	40	40
*Dypsis dracaenoides*	EN		4	4	Described post-1995	0	0
*Dypsis dransfieldii*	NT	EN	12	8	Downlisted	100	100
*Dypsis elegans*	CR	CR	1340	27	No change	40	70
*Dypsis eriostachys*	EN	EN	2395	20	No change	40	70
*Dypsis faneva*	EN	EN	2037	94	No change	50	90
*Dypsis fanjana*	EN	EN	10772	110	No change	80	80
*Dypsis fasciculata*	NT	VU	74598	2304	Downlisted	40	70
*Dypsis fibrosa*	LC	NotT	160135	4400	No change	40	60
*Dypsis forficifolia*	LC	NotT	29506	4500	No change	40	80
*Dypsis furcata*	EN	EW	7914	25	Downlisted	0	50
*Dypsis gautieri*	VU	–	6	3	Described post-1995	0	100
*Dypsis glabrescens*	EN	EN	3195	61	No change	60	70
*Dypsis gronophyllum*	CR	–	4	4	Described post-1995	0	0
*Dypsis henrici*	DD	DD	–	–	No change	–	–
*Dypsis heteromorpha*	DD	DD	–	–	No change	–	–
*Dypsis heterophylla*	NT	Rare	91693	1225	Not comparable	40	60
*Dypsis hiarakae*	VU	VU	17316	700	No change	70	90
*Dypsis hildebrandtii*	NT	VU	22794	1700	Downlisted	40	60
*Dypsis hovomantsina*	CR	CR	5225	288	No change	60	100
*Dypsis humbertii*	VU	VU	10780	810	No change	40	80
*Dypsis humilis*	CR	–	4	4	Described post-1995	0	0
*Dypsis ifanadianae*	CR	CR	25	6	No change	0	0
*Dypsis integra*	EN	CR	35824	294	Downlisted	40	70
*Dypsis intermedia*	CR	CR	4	4	No change	100	100
*Dypsis interrupta*	CR	CR	378	10	No change	40	60
*Dypsis jeremiei*	CR	–	4	4	Described post-1995	100	100
*Dypsis jumelleana*	VU	VU	15117	1100	No change	40	40
*Dypsis laevis*	CR	CR	4	4	No change	100	100
*Dypsis lantzeana*	VU	VU	12141	2925	No change	50	70
*Dypsis lanuginosa*	CR	EW	18	18	Downlisted	50	50
*Dypsis lastelliana*	LC	NotT	72396	2340	No change	50	60
*Dypsis leptocheilos*	CR	DD	4	4	Not comparable	0	0
*Dypsis ligulata*	DD	EW	–	–	Not comparable	–	–
*Dypsis linearis*	EN	EW	153	96	Downlisted	50	50
*Dypsis lokohoensis*	VU	VU	6506	600	No change	40	100
*Dypsis loucoubensis*	CR	EN	367	100	Uplisted	100	100
*Dypsis louvelii*	VU	VU	8884	612	No change	20	60
*Dypsis lucens*	DD	EW	–	–	Not comparable	–	–
*Dypsis lutea*	EN	CR	1435	90	Downlisted	30	30
*Dypsis lutescens*	NT	NotT	51777	1700	Uplisted	20	20
*Dypsis madagascariensis*	LC	Rare	115274	5175	Not comparable	30	40
*Dypsis mahia*	CR	CR	4	4	No change	100	100
*Dypsis makirae*	VU	–	18	12	Described post-1995	0	80
*Dypsis malcomberi*	EN	VU	615	64	Uplisted	100	100
*Dypsis mananjarensis*	NT	VU	25568	1200	Downlisted	20	30
*Dypsis mangorensis*	CR	CR	4000	80	No change	40	40
*Dypsis marojejyi*	VU	VU	337	21	No change	100	100
*Dypsis mcdonaldiana*	EN	VU	3835	48–499	Uplisted	40	70
*Dypsis metallica*	CR	–	4	4	Described post-1995	100	100
*Dypsis minuta*	VU	VU	127	45	No change	80	80
*Dypsis mirabilis*	EN	EN	267	102	No change	70	70
*Dypsis mocquerysiana*	NT	VU	7596	3145	Downlisted	60	70
*Dypsis monostachya*	DD	DD	492	–	No change	–	–
*Dypsis montana*	VU	DD	52	14	Not comparable	100	100
*Dypsis moorei*	EN	EN	4623	25	No change	50	50
*Dypsis nauseosa*	CR	CR	4295	256	No change	0	50
*Dypsis nodifera*	LC	NotT	162112	6400	No change	50	80
*Dypsis nossibensis*	CR	CR	4	4	No change	100	100
*Dypsis occidentalis*	VU	DD	9567	600	Not comparable	60	60
*Dypsis onilahensis*	VU	VU	225319	4950	No change	40	40
*Dypsis oreophila*	VU	VU	19830	1000	No change	30	80
*Dypsis oropedionis*	CR	CR	5431	120	No change	30	30
*Dypsis ovobontsira*	CR	CR	4	4	No change	100	100
*Dypsis pachyramea*	LC	VU	883	279	Downlisted	70	70
*Dypsis paludosa*	VU	VU	19094	1452	No change	40	70
*Dypsis perrieri*	VU	VU	23202	540	No change	60	80
*Dypsis pervillei*	CR	EW	2892	4	Not comparable	30	30
*Dypsis pilulifera*	VU	VU	68666	704	No change	60	90
*Dypsis pinnatifrons*	LC	NotT	250579	6000	No change	30	80
*Dypsis plumosa*	DD	–	–	–	Described post-1995	–	–
*Dypsis plurisecta*	DD	EW	–	–	Not comparable	–	–
*Dypsis poivreana*	EN	CR	289	30	Downlisted	0	100
*Dypsis prestoniana*	VU	VU	15208	400	No change	30	80
*Dypsis procera*	VU	VU	18576	756	No change	40	70
*Dypsis procumbens*	NT	NotT	161478	5746	Uplisted	50	70
*Dypsis psammophila*	EN	CR	4234	112	Downlisted	0	90
*Dypsis pulchella*	CR	EW	11766	147	Not comparable	0	0
*Dypsis pumila*	CR	VU	4	4	Uplisted	100	100
*Dypsis pusilla*	VU	VU	2212	342	No change	100	100
*Dypsis rakotonasoloi*	CR	–	6	4	Described post-1995	0	100
*Dypsis ramentacea*	CR	CR	4	4	No change	100	100
*Dypsis reflexa*	CR	–	6	4	Described post-1995	100	100
*Dypsis remotiflora*	CR	EW	5045	14	Not comparable	50	50
*Dypsis rivularis*	EN	VU	16789	457	Uplisted	60	60
*Dypsis robusta*	CR	–	4	4	Described post-1995	0	0
*Dypsis sahanofensis*	CR	EN	16653	45	Uplisted	0	0
*Dypsis saintelucei*	EN	CR	22453	210	Downlisted	0	80
*Dypsis sancta*	CR	–	4	4	Described post-1995	100	100
*Dypsis sanctaemariae*	CR	CR	7	7	No change	0	0
*Dypsis scandens*	CR	CR	15	7	No change	0	0
*Dypsis schatzii*	EN	VU	108	18	Uplisted	100	100
*Dypsis scottiana*	VU	VU	7612	1529	No change	40	40
*Dypsis serpentina*	VU	VU	901	216	No change	40	80
*Dypsis simianensis*	EN	EN	25806	340	No change	40	70
*Dypsis singularis*	CR	CR	19	4	No change	50	50
*Dypsis soanieranae*	DD	EW	–	–	Not comparable	–	–
*Dypsis spicata*	LC	Rare	24904	1300	Not comparable	40	70
*Dypsis tanalensis*	CR	EW	8	8	Not comparable	0	0
*Dypsis tenuissima*	EN	EN	1242	64	No change	50	100
*Dypsis thermarum*	VU	Rare	77	72	Not comparable	80	100
*Dypsis thiryana*	VU	Rare	62521	1245	Not comparable	60	70
*Dypsis thouarsiana*	DD	DD	–	–	No change	–	–
*Dypsis tokoravina*	CR	EN	341	97	Uplisted	70	100
*Dypsis trapezoidea*	CR	CR	4	4	No change	0	0
*Dypsis tsaratananensis*	DD	DD	8	–	No change	–	–
*Dypsis tsaravoasira*	VU	EN	40289	891	Downlisted	70	80
*Dypsis turkii*	EN	–	1602	462	Described post-1995	70	70
*Dypsis utilis*	EN	VU	11592	440	Uplisted	70	90
*Dypsis viridis*	VU	VU	6893	962	No change	60	80
*Dypsis vonitrandambo*	CR	–	8	8	Described post-1995	100	100
*Lemurophoenix halleuxii*	EN	EN	1729	31	No change	50	70
*Marojejya darianii*	EN	CR	11080	80	Downlisted	50	80
*Marojejya insignis*	LC	VU	91513	2710	Downlisted	60	70
*Masoala kona*	EN	EN	693	36	No change	0	30
*Masoala madagascariensis*	CR	VU	15803	77	Uplisted	40	80
*Orania longisquama*	LC	Rare	151841	2345	Not comparable	30	80
*Orania ravaka*	VU	VU	8913	220	No change	70	80
*Orania trispatha*	VU	CR	25198	1644	Downlisted	70	90
*Ravenea albicans*	EN	EN	19384	929	No change	80	90
*Ravenea beentjei*	CR	–	7	5	Described post-1995	0	100
*Ravenea delicatula*	CR	–	6	4	Described post-1995	0	0
*Ravenea dransfieldii*	EN	VU	37979	1856	Uplisted	80	90
*Ravenea glauca*	VU	VU	9989	443	No change	100	100
*Ravenea hypoleuca*	CR	–	575	8	Described post-1995	0	0
*Ravenea julietiae*	EN	EN	34734	112	No change	40	80
*Ravenea krociana*	EN	VU	10241	450	Uplisted	70	70
*Ravenea lakatra*	VU	VU	44	9	No change	40	70
*Ravenea latisecta*	CR	EN	44	9	Uplisted	50	100
*Ravenea louvelii*	CR	EN	9	4	Uplisted	80	80
*Ravenea madagascariensis*	LC	Rare	137043	45300	Not comparable	60	70
*Ravenea musicalis*	VU	VU	4	4	No change	0	0
*Ravenea nana*	EN	EN	75260	220	No change	40	40
*Ravenea rivularis*	EN	VU	2122	144	Uplisted	20	20
*Ravenea robustior*	NT	Rare	312828	2200	Not comparable	60	70
*Ravenea sambiranensis*	LC	VU	355990	52360	Downlisted	60	60
*Ravenea xerophila*	VU	EN	17191	676	Downlisted	30	40
*Satranala decussilvae*	EN	EN	3248	86	No change	50	80
*Tahina spectabilis*	CR	–	4	4	Described post-1995	0	0
*Voanioala gerardii*	CR	CR	264	12	No change	60	80

Where available, both the 1995 and 2012 conservation assessments are given, along with EOO (extent of occurrence) and AOO (area of occupancy) from the 2012 assessment; see methods for details. The percentage of populations (geographically distinct groups [30]) recorded inside the MNP and SAPM protected area networks is also given for each species (note that the expanded SAPM network includes MNP). IUCN Red List categories: Extinct in the Wild (EW), Critically Endangered (CR), Endangered (EN), Vulnerable (VU), Near Threatened (NT), Least Concern (LC), Data Deficient (DD) [30]; two additional ratings were used in the 1995 assessment [10], Not Threatened (NotT) and Rare.

### Comparison with 1995 assessments

**Figure 3:**
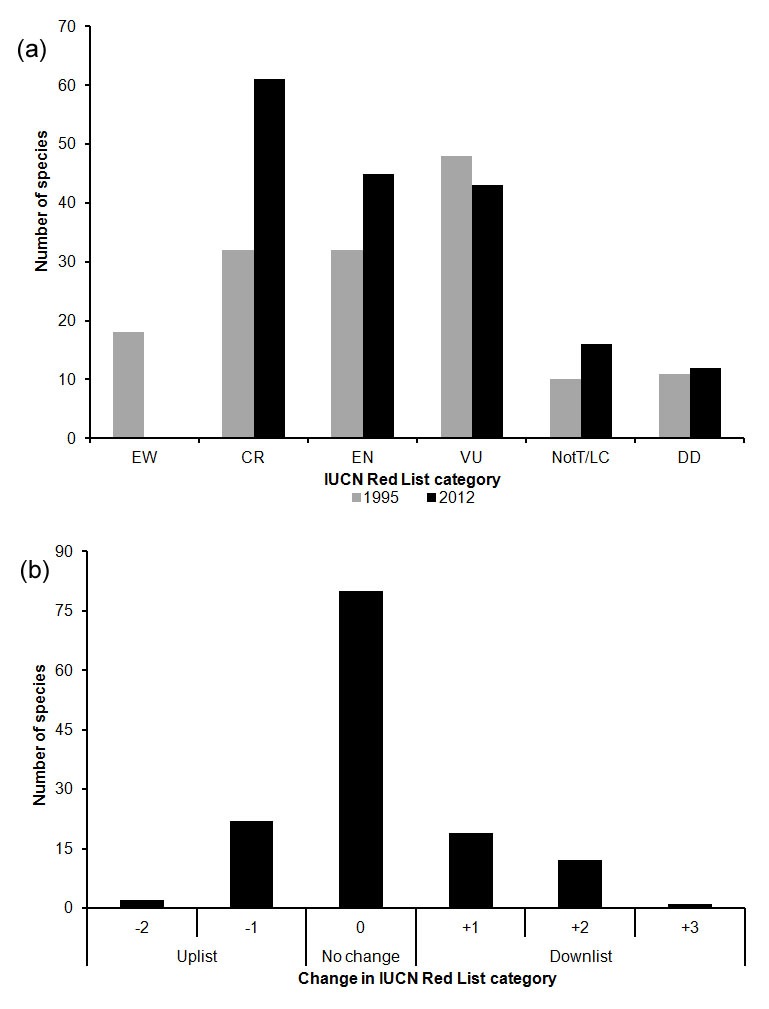
Comparison between the palm assessment of 1995 and 2012. IUCN Red List categories: Extinct in the Wild (EW), Critically Endangered (CR), Endangered (EN), Vulnerable (VU), Least Concern (LC), Data Deficient (DD) [31]. (a) Number of species assessed in each category (total assessments: 164 in 1995, 192 in 2012). All species assessed in each year are illustrated (see Table 3), except for those placed in categories that are not comparable (13 species assessed as “Rare” in 1995 [10] and 14 species assessed as NT in 2012 [44]; see methods). (b) Change in IUCN Red List status (see Table 3) where positive values indicate downlisting to lower extinction risk (e.g. CR to EN is a downlisting of 1 step) and negative values indicate uplisting to higher extinction risk (e.g. EN to CR is an uplisting of 1 step). Figure 3b includes 130 data sufficient species (i.e. excluding species rated as DD in either year) that were assessed in comparable categories in both 1995 [10] and 2012 [44].

Comparison between the 1995 assessments and the 2012 assessments is complicated due to the change in Red List criteria from earlier versions to version 3.1 [29]–[31], improved knowledge of species distributions and changes to the overall taxonomy of Madagascar palms due to many new species being described after 1995. Consequently, a Red List Index approach [53], [54] was not used here. Figure 3a illustrates numbers of species assessed in each of the IUCN Red List categories for categories that are comparable in both the 1995 assessment and the 2012 assessments (i.e. excluding NT and “Rare”). Numbers of species assessed as DD, LC and VU were similar in both assessments (11, 10 and 48 in 1995, 13, 16 and 43 in 2012, respectively; Fig. 3a). In contrast, numbers of species assessed as EN and CR were much higher in 2012 than 1995 (32 and 32 in 1995, 45 and 61 in 2012, respectively; Fig. 3a). There are two main reasons for this. Firstly, all 18 species assessed as EW in 1995 were assigned to other categories in 2012 based on additional information, the majority being rated as EN or CR. Five of the 18 species were assessed as DD, but insufficient evidence was found to rate any species as EW in the 2012 assessment. Secondly, 28 species were discovered and described after 1995 and were thus assessed for the first time in 2012. Of these species, only *Dypsis ankirindro* (NT) is not regarded as threatened while the remainder are most being classified as CR (18 species). These newly discovered species are mostly known only from a single site where area of occupancy (AOO) is often low (<4 km^2^, e.g. *Dypsis andilamenensis*, *D. gronophyllum*, *Tahina spectabilis*) and known population sizes are small, some with less than 10 mature individuals recorded in the wild (e.g. *Dypsis humilis*, *D. robusta*).

Comparison of the classes of change between 1995 and 2012 reveals a contrasting pattern (Fig. 3b). Of the 130 species evaluated in both years with data sufficient, comparable assessments, most (80) species showed no change in status and more species were downlisted than uplisted. Specifically, 32 species moved from EW to CR, EN or VU, CR to EN or VU, and EN to VU or NT, and 24 species moved from EN to CR, VU to EN or CR, and NotT to NT. The downlisting of species is primarily due to increased knowledge of palm distributions and populations, rather than an actual change in their threat status in the wild. Changes in status may also be partly due to criteria being more rigorously applied in 2012. However, deforestation and over-exploitation of some species has resulted in the genuine decline of populations or even to local extinction (e.g. *Dypsis ambositrae*, *D. ifanadianae* and *Voanioala gerardii*), and has resulted in genuine uplisting of these taxa.

### Protected area coverage

The expansion of protected areas in Madagascar from the older MNP network to the new SAPM network is highly beneficial to palms. Under the MNP network, 56 species were not included in protected areas. The SAPM network protects at least one population of all but 28 species, a significant improvement over the MNP Network (Tables 3 and 4). Moreover, the SAPM network protects many additional populations of threatened palm species that were not protected by the MNP network. In total, the protected area coverage of populations of 77 threatened species is increased, with an average of 42% more populations being protected under the SAPM network than MNP for these species (Table 3).

**Table 4:** Threatened and data deficient palm species that do not occur in the SAPM protected area network.

Species	Location	Major threats
*Beccariophoenix alfredii* (VU)	Betafo	Fires, harvest of seeds for horticulture
*Dypsis andilamenensis* (CR)	Andilamena	Habitat loss due to mining and agriculture
*Dypsis ankaizinensis* (DD)	Tsaratanana	Unknown
*Dypsis aquatilis* (CR)	Fort-Dauphin	Fire, habitat loss due to mining and agriculture
*Dypsis basilonga* (EN)	Andrambovato and Vatovavy	Habitat loss due agriculture, harvest of seeds for horticulture, harvest of palm heart
*Dypsis canescens* (DD)	Ambilobe	^Unknown^
*Dypsis commersoniana* (DD)	Fort-Dauphin	Unknown
*Dypsis dracaenoides* (CR)	Vondrozo	Habitat loss due to logging and agriculture
*Dypsis gronophyllum* (CR)	Vondrozo	Habitat loss due to logging and agriculture
*Dypsis henricii* (DD)	Fort-Dauphin	Unknown
*Dypsis heteromorpha* (DD)	Tsaratanana	Unknown
*Dypsis humilis* (CR)	Ambodivoahangy (Makira)	Habitat loss due to logging and agriculture
*Dypsis ifanadianae* (CR)	Ifanadiana	Habitat loss due to logging and agriculture, harvest of seeds for horticulture
*Dypsis leptocheilos* (CR)	Maevatanana	Habitat loss due to agriculture, harvest of seeds for horticulture
*Dypsis ligulata* (DD)	Ambilobe	Unknown
*Dypsis monostachya* (DD)	Maroantsetra	Unknown
*Dypsis plurisecta* (DD)	Maroantsetra	Unknown
*Dypsis pulchella* (CR)	Andilamena	Habitat loss due to mining, logging and agriculture
*Dypsis robusta* (CR)	Ifanadiana	Habitat loss due to agriculture
*Dypsis sahanofensis* (CR)	Ambositra, Anosibe an’Ala and Vatovavy	Habitat loss due to logging and agriculture
*Dypsis sanctaemariae* (CR)	Sainte Marie	Habitat loss due to logging and agriculture
*Dypsis scandens* (CR)	Ifanadiana	Habitat loss due to logging and agriculture, harvest of stems for weaving
*Dypsis soanieranae* (DD)	Soanierana Ivongo	Unknown
*Dypsis tanalensis* (CR)	Vondrozo	Habitat loss due to logging and agriculture
*Dypsis trapezoidea* (CR)	Vatovavy	Habitat loss due to logging and agriculture
*Ravenea delicatula* (CR)	Andilamena	Habitat loss due to mining, logging and agriculture
*Ravenea musicalis* (CR)	Fort-Dauphin	Harvest of seeds for horticulture, harvest of stems to make canoes
*Tahina spectabilis* (CR)	Analalava (Mahajanga)	Fire, grazing by livestock

Three remaining data deficient species are not listed here as their distributions are unknown (Dypsis lucens, D. plumosa and D. thouarsiana). Some of the locations listed here are close to protected areas (e.g. Tsaratanana, Vondrozo), but the known palm localities fall outside the protected areas boundaries.

**Figure 4:**
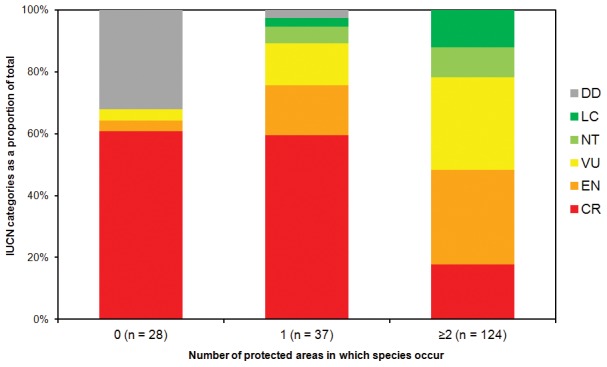
IUCN conservation status of palm species summarised by occurrence in protected areas (SAPM network). Of the 192 assessed species [44], 28 are not recorded from any protected area (coded as 0 in the figure), 37 species are recorded only from one protected area (coded as 1) and 124 species occur in two or more protected areas (coded as ≥2). Three data deficient species are not included as their distributions are unknown (Dypsis lucens, D. plumosa and D. thouarsiana).

Comparison of IUCN Red List assessments with species presence in protected areas (Fig. 4) demonstrates that species known only from outside the SAPM network are either threatened or DD (Table 4). All have small range sizes, many persist in degraded habitats and some have not been seen in the wild for several decades. The majority of the unprotected, threatened species are assessed as CR, e.g. *Dypsis ifanadianae*, *D. scandens* and *Ravenea musicalis*. Some occur in forested areas unconnected to the protected area network (e.g. Ambilobe, Ifanadiana, Vatovavy), whereas others occur in forest adjacent to protected area boundaries (e.g. Andilamena, Tsaratanana, Vondrozo).

Of the species that are protected within the SAPM network, 37 have been recorded in only one protected area while 124 have been documented in two or more protected areas (Fig. 4). The most important protected areas for palms are Masoala, Makira (both 43 species) and Mananara Avaratra (41 species). Marojejy, the Fandriana-Vondrozo Corridor, Manompana, Betampona and Mangerivola each contain more than 20 species. It is significant that three of these eight palm hotspots are newly designated protected areas (the Fandriana-Vondrozo Corridor, Makira, Manompana), further emphasising the importance of the expanded SAPM network. These protected areas vary widely in extent, but all are located in the humid forested east. Nevertheless, protected areas do not guarantee low extinction risk as the majority of species that occur in protected areas are still assessed as threatened (Fig. 4), indicating that threatening processes persist in these areas.

### Threats

**Figure 5:**
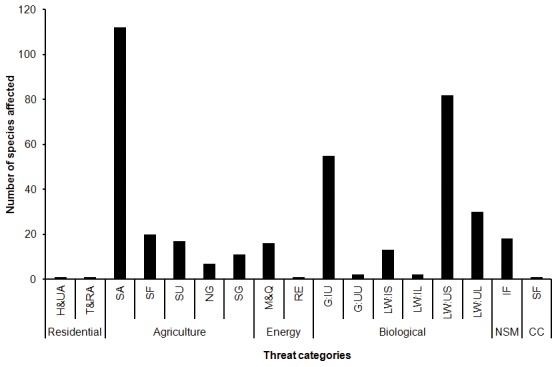
Major threats affecting endemic palm species in Madagascar. Bar heights reflect number of species affected by each threat, as indicated in the 2012 IUCN conservation assessment [44]. Threat categories follow the Threats Classification Scheme version 3.2 [51], using the top two levels of the hierarchy. Abbreviations: Residential & commercial development (Residential): Housing & urban areas (H&UA), Tourism & recreation areas (T&RA); Agriculture & aquaculture (Agriculture): Shifting agriculture (SA), Small-holder farming (SF), Scale unknown/unrecorded (SU), Nomadic grazing (NG), Small-holder grazing, ranching or farming (SG); Energy production & mining (Energy): Mining & quarrying (M&Q), Renewable energy (RE); Biological resource use (Biological): Gathering terrestrial plants, Intentional use (species being assessed is the target) (G:IU), Gathering terrestrial plants, unintentional use (G:UU), Logging & wood harvesting for subsistence, Intentional use: subsistence/small scale (species being assessed is the target) (LW: IS), Logging & wood harvesting, Intentional use: large scale (species being assessed is the target) (LW: IL); Logging & wood harvesting, Unintentional effects: subsistence/small scale (species being assessed is not the target) (LW: US), Logging & wood harvesting, Unintentional effects: large scale (species being assessed is not the target) (LW: UL); Natural system modifications (NSM): Increase in fire frequency (IF); Climate change & severe weather (Climate): Storm & flooding (SF).

**Figure 6:**
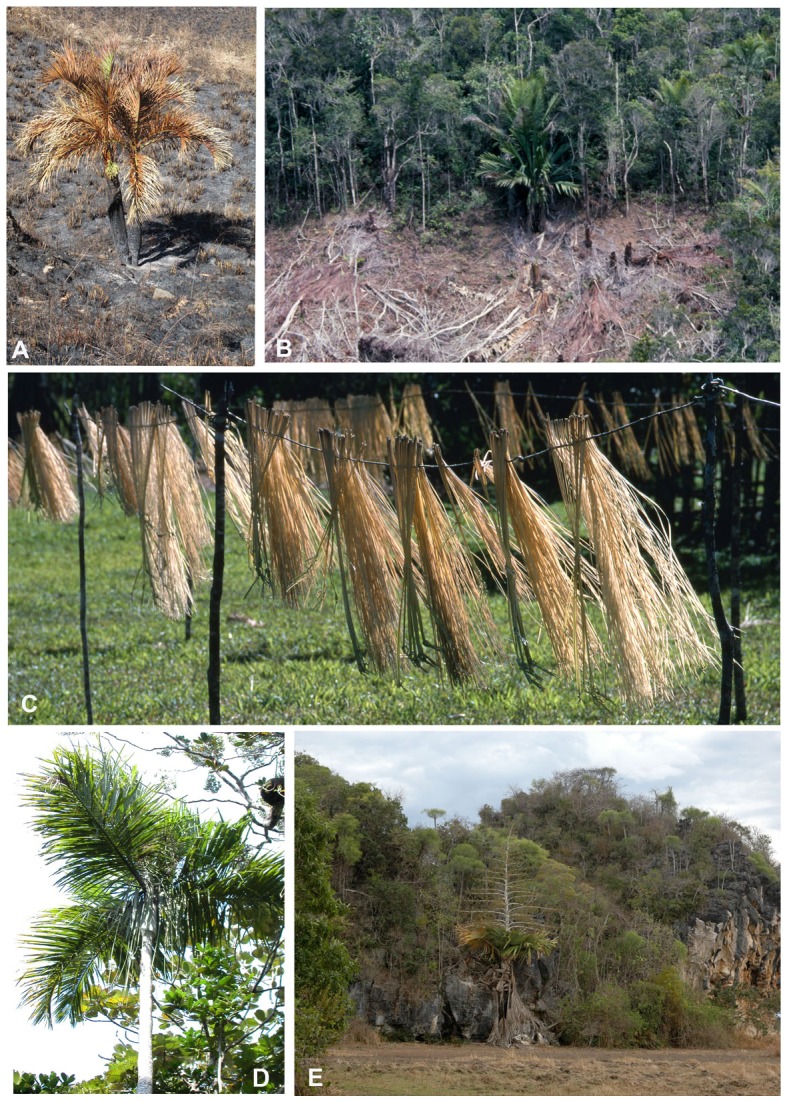
Example of palm species under threat in Madagascar. (a) Anthropogenic fires in grasslands, causing decline and destruction of palm populations, such as *Dypsis decipiens* (VU), Itremo. (b) Forest clearance for slash and burn cultivation by smallholder farmers, causing habitat loss for many species, such as *Masoala kona* (EN), Ifanadiana. (c) Gathering of young leaves of *Ravenea lakatra* (VU) for production of woven hats and basketry, Masoala. (d) Destructive harvest of palm heart threatens many species such as *Dypsis saintelucei* (EN), Sainte Luce. (e) Remnant populations of species such as *Tahina spectabilis* (CR), Analalava, near Mahajanga in vegetation remnants isolated within anthropogenic landscapes, at risk from fire, grazing and other human pressures. Image credits: (a) M Rakotoarinivo, (b) WJ Baker, (c, d & e) J Dransfield.

The major threatening processes for palms in Madagascar are agriculture and biological resource use with 167 and 184 species affected by these threats respectively. More specifically the threats to palm habitats from agriculture relate to annual and perennial non-timber crop production i.e. crops planted for food, fodder, fibre, fuel or other uses, with ‘shifting agriculture’ listed as the scale of farming affecting the highest number of species (112) (Figs. 5 and 6; threat wordings according to IUCN Threats Classification Scheme (version 3.2) [51]). The threat from biological resource use is related to the gathering of terrestrial plants (55 species, e.g. for palm heart consumption) and logging and wood harvesting (127 species). More specifically the highest scoring threat is from logging and wood harvesting for subsistence on a large scale where the species of palm is actually not intended target, but is threatened due to collateral damage (112 species) i.e. the palms are subject to collateral damage. Other less prevalent threats relate to mining, livestock farming, fires, housing and urban development.

## Discussion

As of 2013, 684 native plant species from Madagascar (out of an estimated total of ca. 13,000 [55]) were completely assessed under the IUCN categories and criteria [31] and displayed on the website of the Red List [44]. With 192 species and representing nearly 30% of the Madagascar plant species on the Red List, our study of palms is the largest and most complete IUCN conservation assessment of any plant family on the island. Recently, the IUCN Madagascar Plant Specialist Group (Groupe des Spécialistes des Plantes Malgaches, GSPM) [55] assessed ca. 3,000 plant species from 74 families and 285 genera. In addition, full assessments of smaller taxonomic groups have been completed: Pandanaceae (91 species [25]), Sarcolaenaceae (68 species) and Sphaerosepalaceae (20 species) [56], tribe Coleeae of Bignoniaceae (67 species [57]) and the genus *Delonix* (Fabaceae, 11 species; [58]). Unfortunately, none of these groups of assessments has been formally published on the IUCN Red List yet.

Our finding that as many as 83% of palm species are threatened (best estimate) indicates that the Arecaceae is among the organismal groups facing the highest risk of extinction in Madagascar. Moreover, the proportion of threatened palms in Madagascar is almost four times greater than for plants in general, estimated as 21.5% worldwide [59], and is higher than the estimate for Madagascar’s flora as a whole (54%, [60]). At family level, the percentage of threatened species is variable, 49% of all endemic legumes [57], 65% for Sphaerosepalaceae and 75% for Sarcolaenaceae [56], and 81.3% for Pandanaceae [25]. It is notable that the proportions of threatened species in the Arecaceae and Pandanaceae are similar, given that they share functional similarities as woody, often arborescent monocotyledonous plants that are most species rich in humid forests. For major groups of animals in Madagascar, the proportion of threatened species varies widely, for example, 25% for amphibians [61], 37% for reptiles [60] and 94% for lemurs [62].

Comparisons between the 1995 and 2012 assessments of Madagascar palms [10] indicates that downlisting (a movement to a category of lower threat) is more frequent than uplisting, though 21 of 32 downlisted species still fall into threatened categories. These changes to a lower category come from improved knowledge of species distribution, population size, and taxonomy rather than any genuine decline of extinction risk in the wild. In contrast, almost all of our recently discovered species (species not known to science in 1995) are threatened as they typically have small range sizes and are at risk of habitat loss and direct or indirect threats from human pressure [63]. The role of the taxonomist in conservation assessment cannot be over-stated as collections-based research and knowledge both in the field and in the herbarium or museum is essential for confirming the identity of species and the distribution of their wild populations. Conservation assessment in the absence of robust taxonomy will result in inaccurate ratings and a tendency to categorise species as DD [57], [64]. In our case, intensive taxonomic research and field surveys in Madagascar fundamentally underpin our conservation assessments and have led to the rediscovery of species previously thought to be extinct, as well as the discovery of new populations of threatened species and species new to science.

Our analysis of threats facing palms suggests that the dependency of rural people on forested lands for shifting cultivation and their continued unsustainable exploitation of wild forest products such as palms are key drivers of palm extinction risk. This applies even in remote areas where human population density is low [35]. Our analysis also reveals a novel and insidious threat to palms through the logging or harvesting of other plants at a subsistence level that causes collateral damage to palms. Unless the economic circumstances of rural communities change radically, forest resources such as palms will continue to be exploited unsustainably for basic subsistence needs. Economic factors are a primary concern for the conservation of Madagascar palms, as they are for so many other organisms globally.

Time-delayed biodiversity loss [65] is an important consideration for Madagascar palms as many species persist locally as seedlings or juvenile plants after mature trees have been cleared with the forest. Species in decline may survive for a long time before they become extinct if a threshold in the habitat quality is maintained [66]. Without adequate protection and management, these sites are likely to be lost in the future as disturbance and fragmentation provide suitable habitat for invasive secondary species [12], which have negative impacts on native species by depressing the growth rate at various stages of the life cycle [67].

The high degree of extinction risk faced by Madagascar palms calls into question the effectiveness of previous conservation actions on the island. In a period when human population density and pressure on biodiversity are increasing, the long-term success of protected areas is at the heart of potential solutions for palm conservation. By covering 70% of the remaining humid forest in Madagascar [68], the SAPM network is expected to be considerably more effective for species protection in Madagascar compared with the previous, more limited MNP network, as the new set of reserves has been selected to include narrow range taxa [69]. Our analysis demonstrates that SAPM protects threatened palm populations much more effectively than the MNP network. Nevertheless, the SAPM network has limitations. To date, only Makira has been accorded definitive protected area status, whereas the remainder are not yet formally designated [23]. Consequently, critical protection of the forest and its biodiversity is lacking in the majority of the SAPM network.

Moreover, SAPM does not provide complete protection for Madagascar palms as several priority sites (Table 4), typically forest fragments far from protected areas, are not included. Small areas of intact habitat need to be taken into account as they often contain remnant populations of rare and endemic species [70] that are highly susceptible to environmental stochasticity and local extinction [71]. For example, a monotypic genus of massive fan palm, *Tahina spectabilis*, discovered only in 2006 [72], persists in a 160 × 50 m patch of forested tsingy (karst limestone) surrounded by anthropogenic grassland near Mahajanga. The protection of this forest is an urgent priority to conserve this isolated, endemic lineage. Some small fragments are included in the SAPM network, such as a ca. 2 km^2^ tract of degraded coastal plain forest at Analalava (near Foulpointe), north of Toamasina, which is an outstanding palm hotspot containing 25 palm species, including one local endemic and three species known from only one other site each. This small fragment is managed locally by the Missouri Botanical Garden staff who promote the site for ecotourism [73].

Madagascar palms face exceptional levels of extinction risk by both national and global standards. The conservation of keystone species such as palms [9] is of particular importance due to the potential consequences of their extinction to other species. Humans are among the organisms that rely substantially on ecosystem services provided by palms [10], [15], [74], [75]. The engagement of local communities in conservation initiatives will be critical to their success. Given the intensifying pressure from growing human populations, compounded by projected impacts of climate change on species extinction [76], there is now an urgent need for prioritised action for Madagascar palms. The rigorous IUCN conservation assessment described here provides an essential foundation for such a process.
