# Correction: Comprehensive Red List Assessment Reveals Exceptionally High Extinction Risk to Madagascar Palms

**DOI:** 10.1371/journal.pone.0106619

**Published:** 2014-08-25

**Authors:** The PLOS ONE Staff

## Notice of Republication

This article was republished on August 5, 2014 to correct the figure sizing. The publisher apologizes for this error. Please download this article again to view the corrected figures. The originally published, uncorrected article and the republished, corrected article are provided here for reference.

## Supporting Information

File S1 Originally published, uncorrected article.
(PDF)

File S2 Republished, corrected article.
(PDF)
