# Supplementary material for: Correction: Comprehensive Red List Assessment Reveals Exceptionally High Extinction Risk to Madagascar Palms
Source: PLoS One. 2014 Aug 25;9(8):e106619. doi: 10.1371/journal.pone.0106619 (PMC4143401; doi:10.1371/journal.pone.0106619)
Supplement: File S2 — Republished, corrected article. (PDF) [file pone.0106619.s002.pdf]

OPEN ACCESS Freely available online PLOS ONE

Comprehensive Red List Assessment Reveals Exceptionally High Extinction Risk to Madagascar Palms

Mijoro Rakotoarinivo[1]*, John Dransfield[2], Steven P. Bachman[2], Justin Moat[2], William J. Baker[2]

1 Kew Madagascar Conservation Centre, Ambodivoanjo Ivandry, Antananarivo, Madagascar, 2 Royal Botanic Gardens, Kew, Richmond, Surrey, United Kingdom

Abstract

The establishment of baseline IUCN Red List assessments for plants is a crucial step in conservation planning. Nowhere is this more important than in biodiversity hotspots that are subject to significant anthropogenic pressures, such as Madagascar. Here, all Madagascar palm species are assessed using the IUCN Red List categories and criteria, version 3.1. Our results indicate that 83% of the 192 endemic species are threatened, nearly four times the proportion estimated for plants globally and exceeding estimates for all other comprehensively evaluated plant groups in Madagascar. Compared with a previous assessment in 1995, the number of Endangered and Critically Endangered species has substantially increased, due to the discovery of 28 new species since 1995, most of which are highly threatened. The conservation status of most species included in both the 1995 and the current assessments has not changed. Where change occurred, more species have moved to lower threat categories than to higher categories, because of improved knowledge of species and their distributions, rather than a decrease in extinction risk. However, some cases of genuine deterioration in conservation status were also identified. Palms in Madagascar are primarily threatened by habitat loss due to agriculture and biological resource use through direct exploitation or collateral damage. The recent extension of Madagascar's protected area network is highly beneficial for palms, substantially increasing the number of threatened species populations included within reserves. Notably, three of the eight most important protected areas for palms are newly designated. However, 28 threatened and data deficient species are not protected by the expanded network, including some Critically Endangered species. Moreover, many species occurring in protected areas are still threatened, indicating that threatening processes persist even in reserves. Definitive implementation of the new protected areas combined with local community engagement are essential for the survival of Madagascar's palms.

Citation: Rakotoarinivo M, Dransfield J, Bachman SP, Moat J, Baker WJ (2014) Comprehensive Red List Assessment Reveals Exceptionally High Extinction Risk to Madagascar Palms. PLoS ONE 9(7): e103684. doi:10.1371/journal.pone.0103684

Editor: Francisco Moreira, Institute of Agronomy, University of Lisbon, Portugal

Received January 9, 2014; **Accepted** May 9, 2014; **Published** July 30, 2014

Copyright: © 2014 Rakotoarinivo et al. This is an open-access article distributed under the terms of the Creative Commons Attribution License, which permits unrestricted use, distribution, and reproduction in any medium, provided the original author and source are credited.

Funding: This work was funded by the Friends of Kew through the Threatened Plants Appeal and by the Bentham-Moxon Trust at the Royal Botanic Gardens, Kew. The funders had no role in study design, data collection and analysis, decision to publish, or preparation of the manuscript.

Competing Interests: The authors have declared that no competing interests exist.

* Email: mrakotoarinivo.rbgkew@moov.mg

Introduction

Madagascar is one of the World's most threatened biodiversity hotspots [1] because of the high endemism of its biota coupled with widespread habitat degradation, especially in humid forest areas. Despite ongoing scientific studies that have highlighted Madagascar as a place of endemic megadiversity that is facing intensifying extinction risk [2], the island's charismatic flora and fauna remain under immense pressure [3,4]. Conservation baselines are urgently required to demonstrate and strengthen the case for action on the ground.

Palms are among the most conspicuous components of the flora of Madagascar. To date, 195 species in 17 genera are recognized [5,6] with all but three being endemic to the island (98% endemism). The palm flora of Madagascar is outstandingly rich in a global context [7]. Palms inhabit mostly primary vegetation although a few species occur in disturbed areas, such as anthropogenic grassland. Consistent with global patterns of palm distribution, 90% of Madagascar palms are restricted to humid forest [8,9].

Palms are particularly vulnerable to humid forest degradation. In most species, survival and recruitment are reduced when habitat quality declines [10,11] or when habitats become fragmented [12]. The extensive degradation of Madagascar's humid forests, which have been reduced to around 25% of their original extent [13], implies that the island's humid forest-restricted biota, such as palms, are likely to be extremely threatened.

In addition to habitat loss, palms are further threatened by unsustainable, targeted exploitation by humans. Alongside grasses and legumes, palms are among the most important plant families for humans [14], providing numerous useful resources, such as materials for construction or weaving, food, medicine and ornamental plants [15]. Palms play a particularly important role in poorer countries, such as Madagascar, where they have immense economic importance at the village level [10], but they are often destructively harvested, e.g. for palm heart consumption or construction materials. In recent decades, Madagascar palms have also been targeted by plant collectors for introduction to horticultural trade [16]. These human activities place palms at greater risk of extinction than other humid forest groups that are not exploited in this way.

Extinctions at both species and population levels are of concern because unique evolutionary history and ecosystem services may be lost [17], which is particularly significant in the case of keystone groups such as palms [9]. To prevent such biodiversity loss in

Madagascar palms, a critical conservation strategy is required to focus attention on conservation priorities, to stimulate necessary actions and to raise public awareness. To take these steps, species of concern must first be properly identified based on sound taxonomy [18] so that accurate and cost-effective conservation management decisions can be made. The conservation performance of protected area networks can be improved with such information. Much of Madagascar's biodiversity is unlikely to survive unless it occurs within protected areas [19,20]. In 2003, the Madagascar government decided to increase the protected areas surface [21] from 1.7 million hectares (3%) [22] to 6 million hectare (10% of the island's surface [23]) as many unprotected areas were found to be critically important for biodiversity [24,25].

The International Union for the Conservation of Nature (IUCN) curates the IUCN Red List of Threatened Species, which is the most comprehensive, objective and authoritative data source on extinction risk in species [26–28]. Through the application of a set of five criteria (e.g. restricted range, declining population), a species can be classified according to its relative risk of extinction. In an earlier assessment of the palms of Madagascar [10], in which previous versions of the IUCN system [29,30] were applied, 113 species were identified as threatened and 18 presumed extinct. In this paper, we present a complete and updated conservation assessment of all palm species in Madagascar using the current IUCN Red List categories and criteria (version 3.1 [31]). This work builds upon a robust taxonomy for the group established in recent years [10,32,33,34] and a comprehensive database of collections and observations from recent field work [6].

The objective of this study is to produce a baseline conservation dataset for palms in Madagascar including taxonomy, species distributions, ecological factors and economic uses. We analyze this dataset to answer the following questions: 1) what is the current extinction risk to Madagascar's endemic palm species, 2) how does current status compare with the previous assessment in 1995, 3) is the existing protected area network effective for palms and 4) what are the major threats to palms?

Methods

Study area

Madagascar is a large tropical island (592,750 km^2) in the Indian Ocean [35] and is the third largest tropical island in the world, after New Guinea and Borneo. The island has a complex landscape [36] and is dominated by mountains running north-south, resulting in a central highland region above 800 m elevation. On the eastern side of the central highland is an escarpment that falls steeply away towards the Indian Ocean, whereas the western side consists of a large plain declining gently to the Mozambique Channel. Due to the impact of the southeastern tradewinds (Alizé) and the northwestern monsoon from the Equator, the eastern region is humid to perhumid, the highlands are relatively temperate, the western region is subhumid to dry, and the far south-west is subarid [37]. Consequently, the island has a great diversity of primary vegetation types, ranging from humid forest to dry spiny forest (Fig. 1a) [13]. Humid forest, the primary habitat of most palms, is restricted to the east and north-west of the island. Of the estimated 21 million inhabitants, nearly 80% live in rural areas [38] and depend on natural resources for their subsistence, contributing to the destruction of Madagascar's forests, which have declined by 40% between 1950 and 2000 alone [39].

Occurrence data

The study is based on a dataset of 2,160 georeferenced occurrence records, derived from herbarium specimens of Madagascar palms in key botanical institutions around the world: AAU, FTG, GE, K, MO, NY, P, TAN, TEF and ZT (herbarium acronyms follow [40]). Collection dates range from 1834 to 2010. Records lacking geographic coordinates on specimen labels were georeferenced using topographic maps, online gazetteers, the Madagascar gazetteer of the Missouri Botanical Garden [41] and online mapping tools such as Google Earth [42].

Of the georeferenced records, 820 (38%) postdate 1995 when the previous conservation assessment was conducted [10] of which 561 (26%) result from our own fieldwork in Madagascar since 1995 (fig. 1a). Building on the robust taxonomic baseline provided by Dransfield and Beentje [10], we have conducted targeted fieldwork in 32 sites (Table 1) across Madagascar between 1995–2010 (Fig. 1a), focusing mostly on primary forest areas far from high human density where the palm flora is rich, but poorly known (Fig. 1b). This fieldwork has substantially improved our understanding of the distribution and the populations of 152 species (78% of the total palm flora). The number of specimen records per species ranges from 1 to 85 (mean: 10 specimens per species) and 107 species are known from fewer than 5 specimen records.

All fieldwork was conducted with prior informed consent of the necessary authorities (Table 1). Permission for all fieldwork activities was obtained from the Ministry of Environment and Forests (Ministère de l'Environnement et des Forêts). Additional permissions were required depending on the status of the area visited. For National Parks and Special Reserves, additional permits were issued by Madagascar National Parks (MNP). For Système des Aires Protégées de Madagascar (SAPM) Reserves, additional fieldwork permission was sought from the specific management authority of each site (Table 1). For Local Community Forests, the local village council (Communauté de Base, COBA) was consulted on arrival. Fieldwork on private lands required permission from the land owners in advance (Table 1). Herbarium specimens from our fieldwork were deposited at the Madagascar national herbarium at Parc Botanique et Zoologique de Tsimbazaza (TAN) and the Royal Botanic Gardens, Kew (K). Additional duplicates, where available, were distributed primarily to the Missouri Botanical Garden (MO) and the Natural History Museum, Paris (P).

IUCN Red List Conservation Assessments

We conducted a complete assessment of the conservation status of all 192 endemic Madagascar palm species using the IUCN Red List categories and criteria, version 3.1 [31] with reference to the latest guidelines [43]. Assessments were independently reviewed and verified by the IUCN Palm Specialist Group Red List Authority and IUCN Red List Unit. They were subsequently published on-line on the IUCN Red List on 17 October 2012 [44]. All assessments are now accessible via the IUCN Red List web portal at www.iucnredlist.org.

Each species was classified according to one of the following IUCN categories: Extinct in the Wild (EW), Critically Endangered (CR), Endangered (EN), Vulnerable (VU), Near Threatened (NT), Least Concern (LC) or Data Deficient (DD). We used data from our palm occurrence dataset to summarise distribution, population size and threats to species in order to apply the quantitative Red List criteria. Although attempts were made to apply all five criteria in the Red List system (A, declining population; B, geographic range size and fragmentation, decline, or fluctuations; C, small population size and fragmentation, decline or fluctuations; D, very small population; E, quantitative analysis of extinction risk), as

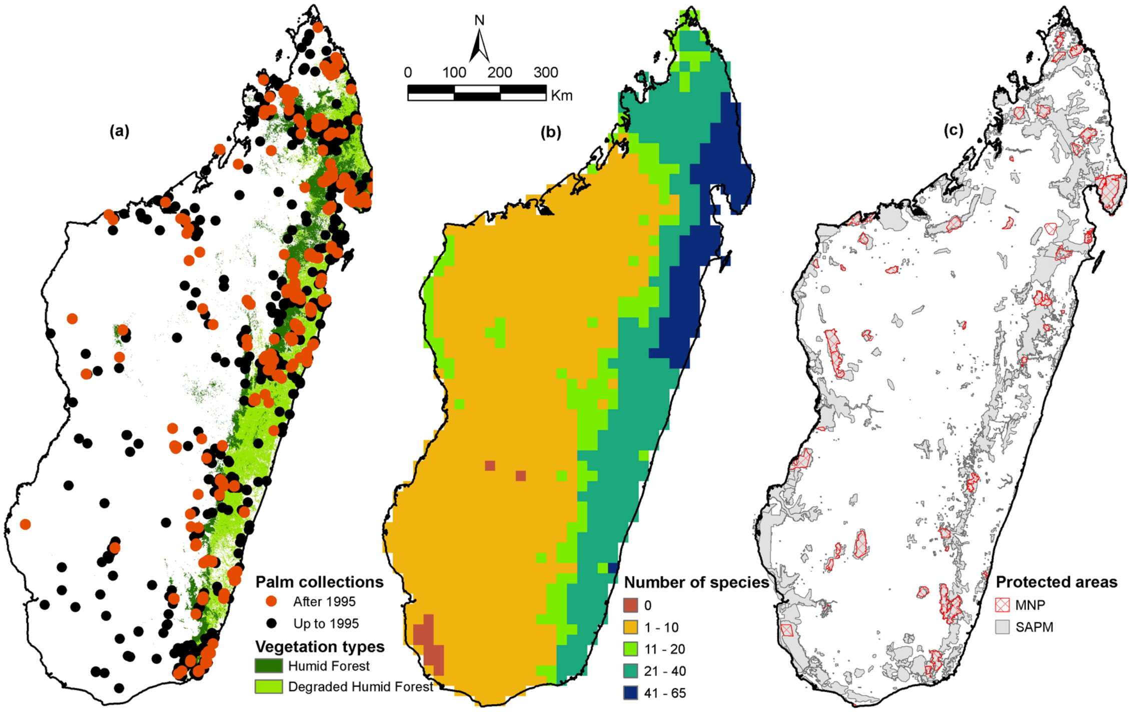

Figure 1. Palm distributions, humid forest and protected areas in Madagascar. (a) Palm specimen collection localities in Madagascar and extent of humid forest vegetation [13]. (b) Species richness of palms in Madagascar [6] illustrating predicted number of palm species across the island at a resolution of 0.2° (ca. 22 km × 22 km). (c) Protected area network in Madagascar comprising the long-standing MNP network (46 parks and reserves [50]) and the newly established SAPM (145 reserves, including those of the MNP network [23]).
doi:10.1371/journal.pone.0103684.g001

recommended by IUCN, most assessments were conducted using criterion B due to the limitations of available data, a common pattern for Red List assessments of plants and some other groups [45].

The palm occurrences were carefully scrutinised for georeference precision, taxonomic identification and likelihood of a population still being extant e.g. historical collections in areas now deforested were excluded. The geographic range of each species was then quantified using two metrics, extent of occurrence (EOO) and area of occupancy (AOO) [31], both of which can be used for assessments under criterion B (restricted range species). EOO was calculated by constructing the minimum convex polygon (convex hull) around known occurrences [46,47] using the Conservation Assessment Tools extension to ArcView [48]. AOO was calculated with the same tools by overlaying a grid and interpreting known occurrences as occupied grid cells. The sum of occupied grid cells equates to the AOO value. A grid cell size of 2×2 km^2 was applied, as recommended by IUCN [43], where sampling effort was deemed sufficient. In some cases, larger cell sizes were used (up to 10×10 km^2 [25,46]) to account for inadequate sampling across the range. These larger grid cells were not scaled down to the reference scale of 2×2 km^2, so the assessments assume the distribution is fully saturated at the 2×2 km^2 reference scale [43]. In cases where a species was known from less than three unique collection sites, EOO could not be calculated and AOO alone was estimated. For species known to occur at a single locality and in a well defined habitat, AOO was estimated by considering the available suitable habitat. Satellite imagery from Google Earth [42] was used to determine suitable areas and polygons were drawn to estimate area of occupancy (AOO).

To infer population trends, such as continuing decline or fragmentation of distribution range through time, GIS layers of the vegetation maps of Humbert & Cours-Darne [49] and Moat & Smith [13] were compared. The rate of the decline of the population of each species in the 42 years between these two baseline vegetation surveys (1965 and 2007) was then calculated from the loss of suitable habitat under its EOO and AOO.

In order to evaluate the trend in conservation status change over time, we compared the 2012 assessment [44] with the previous assessment made by Dransfield & Beentje [10]. The two assessments were based on different versions of the IUCN Red List categories and criteria. The 1995 assessment was broadly based on version 2.3 [29], whereas the 2012 assessment used version 3.1 [31]. While most Red List categories were comparable between the two assessments, the category "Rare" used by Dransfield and Beentje [10] comes from a scheme pre-dating version 2.3 and could not be related to a category in version 3.1. The category "Near Threatened" of version 3.1 was absent from version 2.3. "Not Threatened" (NotT), as used in the 1995 assessment, was regarded as equivalent to "Least Concern" in version 3.1. The change in the IUCN assessments was quantified for data sufficient species (i.e. those that had enough data to carry out a full assessment) that were assessed in both years and in comparable categories. Changes were sorted into three classes: a) no change, if the category of the species was the same in the two assessments, b) downlisted, if the assessment of extinction risk decreased, i.e. from higher to lower category (e.g. EN to VU) and

Table 1. Fieldwork locations visited by the authors.

Location	Latitude and longitude co-ordinates of sites visited	Status
Ambakireny	17.69° S 48.01° E	Local community forest
Ambatovaky	16.86° S 49.26° E	Special Reserve
Ambodivoahangy (Makira)	15.28° S 49.62° E	Local community forest
Analalava (Mahajanga)	14.76 S° 47.43° E	Local community forest
Andilamena	16.98° S 48.84° E; 16.81° S 48.68° E	Local community forest
Anosibe an'Ala	19.66° S 48.11° E	Local community forest
Betafo	20.20° S 46.50° E	Local community forest
Betampona	17.91° S 49.20° E	Special Reserve
Brickaville	18.89° S 49.12° E; 18.96° S 48.85° E	Local community forest
Daraina	13.26° S 49.59° E	SAPM Reserve (managed by Fanamby)
Fenoarivo Atsinanana	17.29° S 49.41° E	SAPM Reserve (managed by Ecole Supérieure des Sciences Agronomiques- Forêts, Antananarivo)
Fort-Dauphin	24.77° S 47.18° E	Private Land (managed by Qit Minerals Madagascar/Rio Tinto)
	24.56° S 47.20° E	SAPM Reserve (managed by Asity Madagascar/Bird Life International)
Analalava (Foulpointe)	17.71° S 49.45° E	SAPM Reserve (managed by Missouri Botanical Gardens)
Ifanadiana	21.33° S 47.71° E	Local community forest
Itremo	20.57° S 46.56° E	SAPM Reserve (managed by Kew Madagascar Conservation Centre)
Maevatanana	16.76° S 47.03° E	Local community forest
Makira	15.38° S 49.44° E; 15.28° S 49.44° E	SAPM Reserve (managed by Wildlife Conservation Society)
Manakara	21.83° S 47.90° E	Local community forest
Mananara Avaratra	15.94° S 49.54° E	Local community forest
Mangerivola	18.20° S 48.92° E	Special Reserve
Mantadia	18.88° S 48.44° E	National Park
Masoala	15.31° S 49.85° E; 15.73° S 49.96° E, 15.74° S 50.19° E, 15.77° S 50.07° E	National Park
Midongy Atsimo	23.55° S 47.08° E	National park
Soanierana Ivongo	16.68° S 49.60° E	Local community forest
Vondrozo	22.80° S 47.18° E	Local community forest

doi:10.1371/journal.pone.0103684.t001

c) uplisted, if the assessment of extinction risk increased, i.e. from lower to higher category (e.g. VU to EN).

Protected area coverage

We compared the distribution of all species to the protected area network in order to assess the effectiveness and coverage of reserves for palm conservation. We used GIS layers (Fig. 1c) describing the 46 established protected areas within the MNP network [50] and the new protected area network being established by SAPM since 2011, which comprises 145 reserves, including those of MNP [23]. We assessed the relative threat status of species occurring in zero, one and two or more protected area to test the expectation that species occurring in fewer protected areas have higher threat ratings.

Threats

During the assessment process the dominant threats for each species were classified according to the IUCN Threats Classification Scheme (version 3.2) [51]. Details about the threats and the local utilization of each species were obtained from expert field observations, specimen labels and literature sources. These data were later compiled to evaluate the relative importance of major threatening processes affecting palms in Madagascar.

Results

IUCN Red List Conservation Assessments

The results of our complete assessment of the conservation status of all known Madagascar palms are summarised in Table 2 and Figure 2, with a detailed break-down given in Table 3. Of the data sufficient species (179), we found that 149 (78%) are classified as threatened (CR, EN or VU). Thirteen species were not data sufficient and were thus rated as DD. Data on the current status of these species were inadequate to complete an assessment primarily because most were known only from the type collection and have not been observed for many years. Taking into account the 13 DD species, we estimated 'lower', 'best estimate' ('mid-point') and 'upper' bounds of the percentage of threatened species [52], which were 78%, 83% and 84% respectively. The lower bound treats all DD species as unthreatened, whereas the upper bound assumes that all are threatened. The best estimate assumes that the same fraction of DD species are threatened as was found for data sufficient species. A total of 14 species were listed as NT, which

Table 2. Summary of results from the 2012 IUCN Red List Assessment of Madagascar Palms.

	Count	Percentage
**IUCN Red List category**		
Extinct (EX)	0	0
Extinct in the Wild (EW)	0	0
Critically Endangered (CR)	61	32
Endangered (EN)	45	23
Vulnerable (VU)	43	22
Near Threatened (NT)	14	7
Least Concern (LC)	16	8
Data Deficient (DD)	13	7
**Summary Statistics**		
Total Evaluated	192	100
Total Data Sufficient (CR+EN+VU+NT+LC)	179	93
**Total Threatened** – lower bound (CR+EN+VU)	149	78
**Total Threatened** – best estimate (mid-point) (CR+EN+VU+((Total Threatened/Data Sufficient) $\times$ DD))	160	83
**Total Threatened** – upper bound (CR+EN+VU+DD)	162	84
Total Species of Elevated Conservation Concern (CR+EN+VU+NT)	163	85
Total Not Threatened (LC+DD)	30	16

doi:10.1371/journal.pone.0103684.t002

gives a total of 163 (91%) species considered to be of elevated conservation concern. Only 16 species were listed as LC.

Comparison with 1995 assessments

Comparison between the 1995 assessments and the 2012 assessments is complicated due to the change in Red List criteria from earlier versions to version 3.1 [29–31], improved knowledge of species distributions and changes to the overall taxonomy of Madagascar palms due to many new species being described after 1995. Consequently, a Red List Index approach [53,54] was not used here. Figure 3a illustrates numbers of species assessed in each of the IUCN Red List categories for categories that are

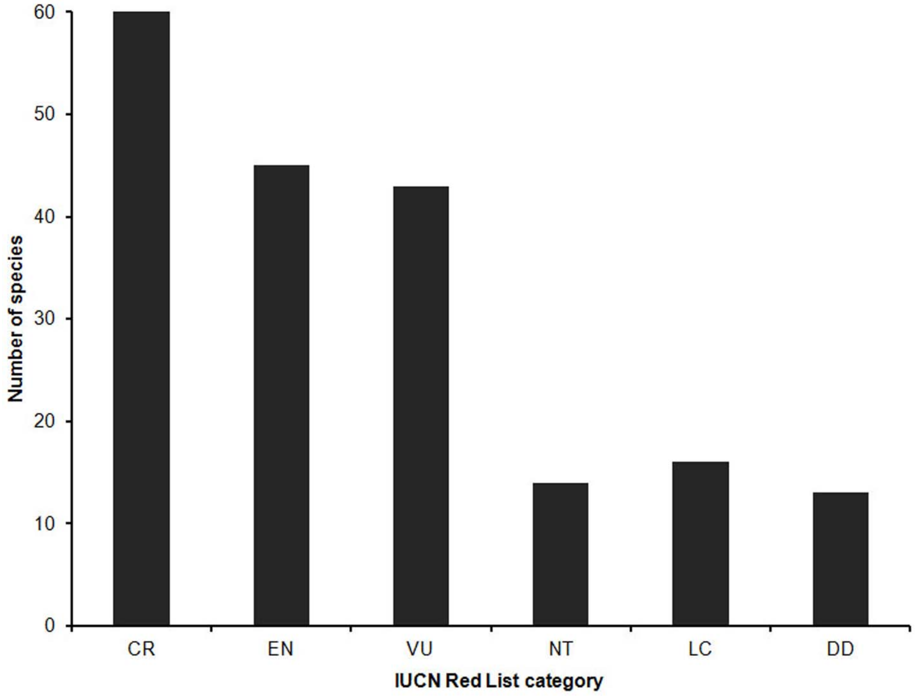

Figure 2. Summary of the 2012 IUCN Red List Assessments of Madagascar Palms (see table 2). IUCN Red List categories: Extinct in the Wild (EW), Critically Endangered (CR), Endangered (EN), Vulnerable (VU), Near Threatened (NT), Least Concern (LC), Data Deficient (DD) [31].
doi:10.1371/journal.pone.0103684.g002

Table 3. The conservation status of all 192 endemic Madagascar palm species.

Species	2012 Red List Assessment	1995 Red List Assessment	EOO (km^2)	AOO (km^2)	Status change	Populations in protected areas: MNP only (%)	Populations in protected areas: SAPM (%)
Beccariophoenix alfredii	VU	–	8	5	Described post-1995	0	0
Beccariophoenix madagascariensis	VU	CR	15460	300	Downlisted	30	80
Bismarckia nobilis	LC	NotT	319415	17100	No change	20	60
Borassus madagascariensis	EN	VU	48872	350	Uplisted	0	40
Dypsis acaulis	EN	EW	72	8	Downlisted	30	30
Dypsis acuminum	EN	DD	12113	150	Not comparable	50	80
Dypsis albofarinosa	CR	–	4	4	Described post-1995	100	100
Dypsis ambanjae	CR	EW	1767	150	Downlisted	50	100
Dypsis ambilaensis	EN	EN	176	60	No change	0	60
Dypsis ambositrae	CR	CR	1790	150	No change	0	30
Dypsis andapae	EN	Rare	1428	35	Not comparable	70	70
Dypsis andilamenensis	CR	–	10	5	Described post-1995	0	0
Dypsis andrianatonga	VU	Rare	5909	288	Not comparable	90	90
Dypsis angusta	EN	EN	5760	123	No change	40	70
Dypsis angustifolia	EN	Rare	469	327	Not comparable	30	100
Dypsis anjae	CR	–	4	4	Described post-1995	100	100
Dypsis ankaizinensis	DD	DD	–	–	No change	–	–
Dypsis ankirindro	NT	–	14	4	Described post-1995	0	100
Dypsis antanambensis	CR	EN	6	6	Uplisted	100	100
Dypsis aquatilis	CR	EN	35	25	Uplisted	0	0
Dypsis arenarum	CR	CR	895	36	No change	0	70
Dypsis baronii	LC	NotT	239065	6075	No change	40	70
Dypsis basilonga	CR	EN	188	16	Uplisted	0	0
Dypsis beentjei	CR	EN	6	6	Uplisted	100	100
Dypsis bejofo	VU	EN	10322	150	Downlisted	60	80
Dypsis bernierana	VU	VU	14610	600	No change	50	60
Dypsis betamponensis	VU	EW	4	4	Downlisted	100	100
Dypsis betsimisarakae	VU	–	7995	512	Described post-1995	50	80
Dypsis boiviniana	EN	EN	7196	896	No change	30	60
Dypsis bonsai	VU	VU	14420	520	No change	80	90
Dypsis bosseri	EN	EW	4	1.5	Downlisted	0	90
Dypsis brevicaulis	CR	CR	380	135	No change	0	80
Dypsis brittiana	CR	–	4	4	Described post-1995	0	100
Dypsis canaliculata	CR	EW	4114	243	Downlisted	30	70
Dypsis canescens	DD	EW	–	–	Not comparable	–	–

Table 3. Cont.

Species	2012 Red List Assessment	1995 Red List Assessment	EOO (km^2)	AOO (km^2)	Status change	Populations in protected areas: MNP only (%)	Populations in protected areas: SAPM (%)
Dypsis carlsmithii	CR	–	2041	8	Described post-1995	50	100
Dypsis catatiana	LC	NotT	146761	3300	No change	70	80
Dypsis caudata	CR	CR	4	4	No change	100	100
Dypsis ceracea	EN	EW	11211	225	Downlisted	40	100
Dypsis commersoniana	DD	CR	–	–	Not comparable	–	–
Dypsis concinna	NT	VU	30591	2100	Downlisted	30	100
Dypsis confusa	NT	Rare	49262	1216	Not comparable	60	70
Dypsis cookei	CR	EN	6	4	Uplisted	100	100
Dypsis coriacea	NT	VU	11642	910	Downlisted	80	90
Dypsis corniculata	EN	VU	18147	1350	Uplisted	40	40
Dypsis coursii	LC	VU	3737	256	Downlisted	70	100
Dypsis crinita	NT	Rare	67442	1134	Not comparable	60	90
Dypsis culminis	EN	–	564	10	Described post-1995	0	100
Dypsis curtisii	EN	DD	7185	64	Not comparable	60	90
Dypsis decaryi	VU	VU	339	83	No change	40	40
Dypsis decipiens	VU	EN	42846	1430	Downlisted	20	40
Dypsis delicatula	VU	–	8	8	Described post-1995	40	80
Dypsis digitata	CR	CR	2334	8	No change	40	40
Dypsis dracaenoides	EN		4	4	Described post-1995	0	0
Dypsis dransfieldii	NT	EN	12	8	Downlisted	100	100
Dypsis elegans	CR	CR	1340	27	No change	40	70
Dypsis eriostachys	EN	EN	2395	20	No change	40	70
Dypsis faneva	EN	EN	2037	94	No change	50	90
Dypsis fanjana	EN	EN	10772	110	No change	80	80
Dypsis fasciculata	NT	VU	74598	2304	Downlisted	40	70
Dypsis fibrosa	LC	NotT	160135	4400	No change	40	60
Dypsis forficifolia	LC	NotT	29506	4500	No change	40	80
Dypsis furcata	EN	EW	7914	25	Downlisted	0	50
Dypsis gautieri	VU	–	6	3	Described post-1995	0	100
Dypsis glabrescens	EN	EN	3195	61	No change	60	70
Dypsis gronophyllum	CR	–	4	4	Described post-1995	0	0
Dypsis henrici	DD	DD	–	–	No change	–	–
Dypsis heteromorpha	DD	DD	–	–	No change	–	–
Dypsis heterophylla	NT	Rare	91693	1225	Not comparable	40	60
Dypsis hiarakae	VU	VU	17316	700	No change	70	90

Table 3. Cont.

Species	2012 Red List Assessment	1995 Red List Assessment	EOO (km^2)	AOO (km^2)	Status change	Populations in protected areas: MNP only (%)	Populations in protected areas: SAPM (%)
Dypsis hildebrandtii	NT	VU	22794	1700	Downlisted	40	60
Dypsis hovomantsina	CR	CR	5225	288	No change	60	100
Dypsis humbertii	VU	VU	10780	810	No change	40	80
Dypsis humilis	CR	–	4	4	Described post-1995	0	0
Dypsis ifanadianae	CR	CR	25	6	No change	0	0
Dypsis integra	EN	CR	35824	294	Downlisted	40	70
Dypsis intermedia	CR	CR	4	4	No change	100	100
Dypsis interrupta	CR	CR	378	10	No change	40	60
Dypsis jeremiei	CR	–	4	4	Described post-1995	100	100
Dypsis jumelleana	VU	VU	15117	1100	No change	40	40
Dypsis laevis	CR	CR	4	4	No change	100	100
Dypsis lantzeana	VU	VU	12141	2925	No change	50	70
Dypsis lanuginosa	CR	EW	18	18	Downlisted	50	50
Dypsis lastelliana	LC	NotT	72396	2340	No change	50	60
Dypsis leptocheilos	CR	DD	4	4	Not comparable	0	0
Dypsis ligulata	DD	EW	–	–	Not comparable	–	–
Dypsis linearis	EN	EW	153	96	Downlisted	50	50
Dypsis lokohoensis	VU	VU	6506	600	No change	40	100
Dypsis loucoubensis	CR	EN	367	100	Uplisted	100	100
Dypsis louvelii	VU	VU	8884	612	No change	20	60
Dypsis lucens	DD	EW	–	–	Not comparable	–	–
Dypsis lutea	EN	CR	1435	90	Downlisted	30	30
Dypsis lutescens	NT	NotT	51777	1700	Uplisted	20	20
Dypsis madagascariensis	LC	Rare	115274	5175	Not comparable	30	40
Dypsis mahia	CR	CR	4	4	No change	100	100
Dypsis makirae	VU	–	18	12	Described post-1995	0	80
Dypsis malcomberi	EN	VU	615	64	Uplisted	100	100
Dypsis mananjarensis	NT	VU	25568	1200	Downlisted	20	30
Dypsis mangorensis	CR	CR	4000	80	No change	40	40
Dypsis marojejyi	VU	VU	337	21	No change	100	100
Dypsis mcdonaldiana	EN	VU	3835	48–499	Uplisted	40	70
Dypsis metallica	CR	–	4	4	Described post-1995	100	100
Dypsis minuta	VU	VU	127	45	No change	80	80
Dypsis mirabilis	EN	EN	267	102	No change	70	70
Dypsis mocquerysiana	NT	VU	7596	3145	Downlisted	60	70

Table 3. Cont.

Species	2012 Red List Assessment	1995 Red List Assessment	EOO (km^2)	AOO (km^2)	Status change	Populations in protected areas: MNP only (%)	Populations in protected areas: SAPM (%)
Dypsis monostachya	DD	DD	492	–	No change	–	–
Dypsis montana	VU	DD	52	14	Not comparable	100	100
Dypsis moorei	EN	EN	4623	25	No change	50	50
Dypsis nauseosa	CR	CR	4295	256	No change	0	50
Dypsis nodifera	LC	NotT	162112	6400	No change	50	80
Dypsis nossibensis	CR	CR	4	4	No change	100	100
Dypsis occidentalis	VU	DD	9567	600	Not comparable	60	60
Dypsis onilahensis	VU	VU	225319	4950	No change	40	40
Dypsis oreophila	VU	VU	19830	1000	No change	30	80
Dypsis oropedionis	CR	CR	5431	120	No change	30	30
Dypsis ovobontsira	CR	CR	4	4	No change	100	100
Dypsis pachyramea	LC	VU	883	279	Downlisted	70	70
Dypsis paludosa	VU	VU	19094	1452	No change	40	70
Dypsis perrieri	VU	VU	23202	540	No change	60	80
Dypsis pervillei	CR	EW	2892	4	Not comparable	30	30
Dypsis pilulifera	VU	VU	68666	704	No change	60	90
Dypsis pinnatifrons	LC	NotT	250579	6000	No change	30	80
Dypsis plumosa	DD	–	–	–	Described post-1995	–	–
Dypsis plurisecta	DD	EW	–	–	Not comparable	–	–
Dypsis poivreana	EN	CR	289	30	Downlisted	0	100
Dypsis prestoniana	VU	VU	15208	400	No change	30	80
Dypsis procera	VU	VU	18576	756	No change	40	70
Dypsis procumbens	NT	NotT	161478	5746	Uplisted	50	70
Dypsis psammophila	EN	CR	4234	112	Downlisted	0	90
Dypsis pulchella	CR	EW	11766	147	Not comparable	0	0
Dypsis pumila	CR	VU	4	4	Uplisted	100	100
Dypsis pusilla	VU	VU	2212	342	No change	100	100
Dypsis rakotonasoloi	CR	–	6	4	Described post-1995	0	100
Dypsis ramentacea	CR	CR	4	4	No change	100	100
Dypsis reflexa	CR	–	6	4	Described post-1995	100	100
Dypsis remotiflora	CR	EW	5045	14	Not comparable	50	50
Dypsis rivularis	EN	VU	16789	457	Uplisted	60	60
Dypsis robusta	CR	–	4	4	Described post-1995	0	0
Dypsis sahanofensis	CR	EN	16653	45	Uplisted	0	0
Dypsis saintelucei	EN	CR	22453	210	Downlisted	0	80

Table 3. Cont.

Species	2012 Red List Assessment	1995 Red List Assessment	EOO (km^2)	AOO (km^2)	Status change	Populations in protected areas: MNP only (%)	Populations in protected areas: SAPM (%)
Dypsis sancta	CR	–	4	4	Described post-1995	100	100
Dypsis sanctaemariae	CR	CR	7	7	No change	0	0
Dypsis scandens	CR	CR	15	7	No change	0	0
Dypsis schatzii	EN	VU	108	18	Uplisted	100	100
Dypsis scottiana	VU	VU	7612	1529	No change	40	40
Dypsis serpentina	VU	VU	901	216	No change	40	80
Dypsis simianensis	EN	EN	25806	340	No change	40	70
Dypsis singularis	CR	CR	19	4	No change	50	50
Dypsis soanieranae	DD	EW	–	–	Not comparable	–	–
Dypsis spicata	LC	Rare	24904	1300	Not comparable	40	70
Dypsis tanalensis	CR	EW	8	8	Not comparable	0	0
Dypsis tenuissima	EN	EN	1242	64	No change	50	100
Dypsis thermarum	VU	Rare	77	72	Not comparable	80	100
Dypsis thiryana	VU	Rare	62521	1245	Not comparable	60	70
Dypsis thouarsiana	DD	DD	–	–	No change	–	–
Dypsis tokoravina	CR	EN	341	97	Uplisted	70	100
Dypsis trapezoidea	CR	CR	4	4	No change	0	0
Dypsis tsaratananensis	DD	DD	8	–	No change	–	–
Dypsis tsaravoasira	VU	EN	40289	891	Downlisted	70	80
Dypsis turkii	EN	–	1602	462	Described post-1995	70	70
Dypsis utilis	EN	VU	11592	440	Uplisted	70	90
Dypsis viridis	VU	VU	6893	962	No change	60	80
Dypsis vonitrandambo	CR	–	8	8	Described post-1995	100	100
Lemurophoenix halleuxii	EN	EN	1729	31	No change	50	70
Marojejya darianii	EN	CR	11080	80	Downlisted	50	80
Marojejya insignis	LC	VU	91513	2710	Downlisted	60	70
Masoala kona	EN	EN	693	36	No change	0	30
Masoala madagascariensis	CR	VU	15803	77	Uplisted	40	80
Orania longisquama	LC	Rare	151841	2345	Not comparable	30	80
Orania ravaka	VU	VU	8913	220	No change	70	80
Orania trispatha	VU	CR	25198	1644	Downlisted	70	90
Ravenea albicans	EN	EN	19384	929	No change	80	90
Ravenea beentjei	CR	–	7	5	Described post-1995	0	100
Ravenea delicatula	CR	–	6	4	Described post-1995	0	0
Ravenea dransfieldii	EN	VU	37979	1856	Uplisted	80	90

Table 3. Cont.

Species	2012 Red List Assessment	1995 Red List Assessment	EOO (km^2)	AOO (km^2)	Status change	Populations in protected areas: MNP only (%)	Populations in protected areas: SAPM (%)
Ravenea glauca	VU	VU	9989	443	No change	100	100
Ravenea hypoleuca	CR	–	575	8	Described post-1995	0	0
Ravenea julietiae	EN	EN	34734	112	No change	40	80
Ravenea krociana	EN	VU	10241	450	Uplisted	70	70
Ravenea lakatra	VU	VU	44	9	No change	40	70
Ravenea latisecta	CR	EN	44	9	Uplisted	50	100
Ravenea louvelii	CR	EN	9	4	Uplisted	80	80
Ravenea madagascariensis	LC	Rare	137043	45300	Not comparable	60	70
Ravenea musicalis	VU	VU	4	4	No change	0	0
Ravenea nana	EN	EN	75260	220	No change	40	40
Ravenea rivularis	EN	VU	2122	144	Uplisted	20	20
Ravenea robustior	NT	Rare	312828	2200	Not comparable	60	70
Ravenea sambiranensis	LC	VU	355990	52360	Downlisted	60	60
Ravenea xerophila	VU	EN	17191	676	Downlisted	30	40
Satranala decussilvae	EN	EN	3248	86	No change	50	80
Tahina spectabilis	CR	–	4	4	Described post-1995	0	0
Voanioala gerardii	CR	CR	264	12	No change	60	80

Where available, both the 1995 and 2012 conservation assessments are given, along with EOO (extent of occurrence) and AOO (area of occupancy) from the 2012 assessment; see methods for details. The percentage of populations (geographically distinct groups [30]) recorded inside the MNP and SAPM protected area networks is also given for each species (note that the expanded SAPM network includes MNP). IUCN Red List categories: Extinct in the Wild (EW), Critically Endangered (CR), Endangered (EN), Vulnerable (VU), Near Threatened (NT), Least Concern (LC), Data Deficient (DD) [30]; two additional ratings were used in the 1995 assessment [10], Not Threatened (NotT) and Rare.

doi:10.1371/journal.pone.0103684.t003

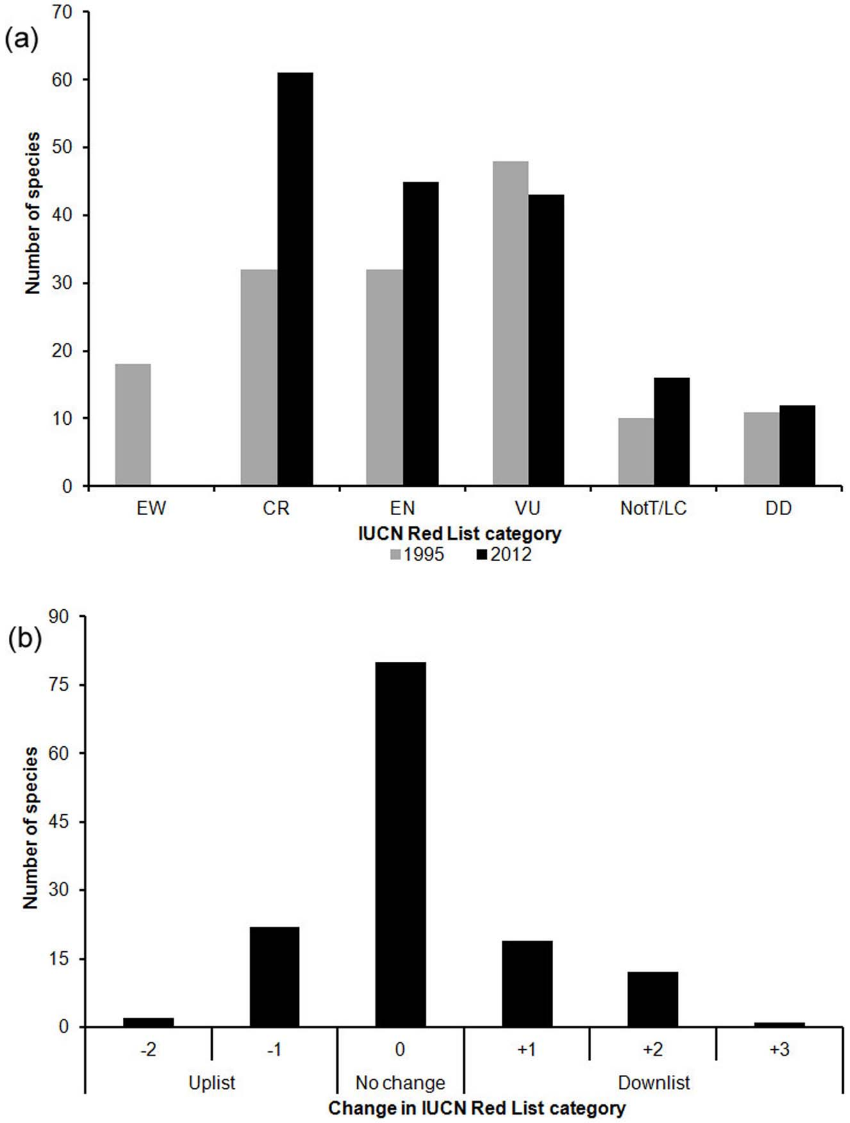

Figure 3. Comparison between the palm assessment of 1995 and 2012. IUCN Red List categories: Extinct in the Wild (EW), Critically Endangered (CR), Endangered (EN), Vulnerable (VU), Least Concern (LC), Data Deficient (DD) [31]. (a) Number of species assessed in each category (total assessments: 164 in 1995, 192 in 2012). All species assessed in each year are illustrated (see Table 3), except for those placed in categories that are not comparable (13 species assessed as "Rare" in 1995 [10] and 14 species assessed as NT in 2012 [44]; see methods). (b) Change in IUCN Red List status (see Table 3) where positive values indicate downlisting to lower extinction risk (e.g. CR to EN is a downlisting of 1 step) and negative values indicate uplisting to higher extinction risk (e.g. EN to CR is an uplisting of 1 step). Figure 3b includes 130 data sufficient species (i.e. excluding species rated as DD in either year) that were assessed in comparable categories in both 1995 [10] and 2012 [44].
doi:10.1371/journal.pone.0103684.g003

comparable in both the 1995 assessment and the 2012 assessments (i.e. excluding NT and "Rare"). Numbers of species assessed as DD, LC and VU were similar in both assessments (11, 10 and 48 in 1995, 13, 16 and 43 in 2012, respectively; Fig. 3a). In contrast, numbers of species assessed as EN and CR were much higher in 2012 than 1995 (32 and 32 in 1995, 45 and 61 in 2012, respectively; Fig. 3a). There are two main reasons for this. Firstly, all 18 species assessed as EW in 1995 were assigned to other categories in 2012 based on additional information, the majority being rated as EN or CR. Five of the 18 species were assessed as DD, but insufficient evidence was found to rate any species as EW in the 2012 assessment. Secondly, 28 species were discovered and described after 1995 and were thus assessed for the first time in 2012. Of these species, only *Dypsis ankirindro* (NT) is not regarded as threatened while the remainder are most being classified as CR (18 species). These newly discovered species are mostly known only from a single site where area of occupancy (AOO) is often low ($<$ 4 km^2, e.g. *Dypsis andilamenensis*, *D. gronophyllum*, *Tahina spectabilis*) and known population sizes are small, some with less than 10 mature individuals recorded in the wild (e.g. *Dypsis humilis*, *D. robusta*).

Comparison of the classes of change between 1995 and 2012 reveals a contrasting pattern (Fig. 3b). Of the 130 species evaluated in both years with data sufficient, comparable assessments, most (80) species showed no change in status and more species were downlisted than uplisted. Specifically, 32 species moved from EW to CR, EN or VU, CR to EN or VU, and EN to VU or NT, and 24 species moved from EN to CR, VU to EN or

Table 4. Threatened and data deficient palm species that do not occur in the SAPM protected area network.

Species	Location	Major threats
Beccariophoenix alfredii (VU)	Betafo	Fires, harvest of seeds for horticulture
Dypsis andilamenensis (CR)	Andilamena	Habitat loss due to mining and agriculture
Dypsis ankaizinensis (DD)	Tsaratanana	Unknown
Dypsis aquatilis (CR)	Fort-Dauphin	Fire, habitat loss due to mining and agriculture
Dypsis basilonga (EN)	Andrambovato and Vatovavy	Habitat loss due agriculture, harvest of seeds for horticulture, harvest of palm heart
Dypsis canescens (DD)	Ambilobe	Unknown
Dypsis commersoniana (DD)	Fort-Dauphin	Unknown
Dypsis dracaenoides (CR)	Vondrozo	Habitat loss due to logging and agriculture
Dypsis gronophyllum (CR)	Vondrozo	Habitat loss due to logging and agriculture
Dypsis henricii (DD)	Fort-Dauphin	Unknown
Dypsis heteromorpha (DD)	Tsaratanana	Unknown
Dypsis humilis (CR)	Ambodivoahangy (Makira)	Habitat loss due to logging and agriculture
Dypsis ifanadianae (CR)	Ifanadiana	Habitat loss due to logging and agriculture, harvest of seeds for horticulture
Dypsis leptocheilos (CR)	Maevatanana	Habitat loss due to agriculture, harvest of seeds for horticulture
Dypsis ligulata (DD)	Ambilobe	Unknown
Dypsis monostachya (DD)	Maroantsetra	Unknown
Dypsis plurisecta (DD)	Maroantsetra	Unknown
Dypsis pulchella (CR)	Andilamena	Habitat loss due to mining, logging and agriculture
Dypsis robusta (CR)	Ifanadiana	Habitat loss due to agriculture
Dypsis sahanofensis (CR)	Ambositra, Anosibe an'Ala and Vatovavy	Habitat loss due to logging and agriculture
Dypsis sanctaemariae (CR)	Sainte Marie	Habitat loss due to logging and agriculture
Dypsis scandens (CR)	Ifanadiana	Habitat loss due to logging and agriculture, harvest of stems for weaving
Dypsis soanieranae (DD)	Soanierana Ivongo	Unknown
Dypsis tanalensis (CR)	Vondrozo	Habitat loss due to logging and agriculture
Dypsis trapezoidea (CR)	Vatovavy	Habitat loss due to logging and agriculture
Ravenea delicatula (CR)	Andilamena	Habitat loss due to mining, logging and agriculture
Ravenea musicalis (CR)	Fort-Dauphin	Harvest of seeds for horticulture, harvest of stems to make canoes
Tahina spectabilis (CR)	Analalava (Mahajanga)	Fire, grazing by livestock

Three remaining data deficient species are not listed here as their distributions are unknown (Dypsis lucens, D. plumosa and D. thouarsiana). Some of the locations listed here are close to protected areas (e.g. Tsaratanana, Vondrozo), but the known palm localities fall outside the protected areas boundaries.
doi:10.1371/journal.pone.0103684.t004

CR, and NotT to NT. The downlisting of species is primarily due to increased knowledge of palm distributions and populations, rather than an actual change in their threat status in the wild. Changes in status may also be partly due to criteria being more rigorously applied in 2012. However, deforestation and over-exploitation of some species has resulted in the genuine decline of populations or even to local extinction (e.g. *Dypsis ambositrae*, *D. ifanadianae* and *Voanioala gerardii*), and has resulted in genuine uplisting of these taxa.

Protected area coverage

The expansion of protected areas in Madagascar from the older MNP network to the new SAPM network is highly beneficial to palms. Under the MNP network, 56 species were not included in protected areas. The SAPM network protects at least one population of all but 28 species, a significant improvement over the MNP Network (Tables 3 and 4). Moreover, the SAPM network protects many additional populations of threatened palm species that were not protected by the MNP network. In total, the protected area coverage of populations of 77 threatened species is increased, with an average of 42% more populations being protected under the SAPM network than MNP for these species (Table 3).

Comparison of IUCN Red List assessments with species presence in protected areas (Fig. 4) demonstrates that species known only from outside the SAPM network are either threatened or DD (Table 4). All have small range sizes, many persist in degraded habitats and some have not been seen in the wild for several decades. The majority of the unprotected, threatened species are assessed as CR, e.g. *Dypsis ifanadianae*, *D. scandens* and *Ravenea musicalis*. Some occur in forested areas unconnected to the protected area network (e.g. Ambilobe, Ifanadiana, Vatovavy), whereas others occur in forest adjacent to protected area boundaries (e.g. Andilamena, Tsaratanana, Vondrozo).

Of the species that are protected within the SAPM network, 37 have been recorded in only one protected area while 124 have been documented in two or more protected areas (Fig. 4). The most important protected areas for palms are Masoala, Makira (both 43 species) and Mananara Avaratra (41 species). Marojejy, the Fandriana-Vondrozo Corridor, Manompana, Betampona and Mangerivola each contain more than 20 species. It is significant that three of these eight palm hotspots are newly designated protected areas (the Fandriana-Vondrozo Corridor, Makira,

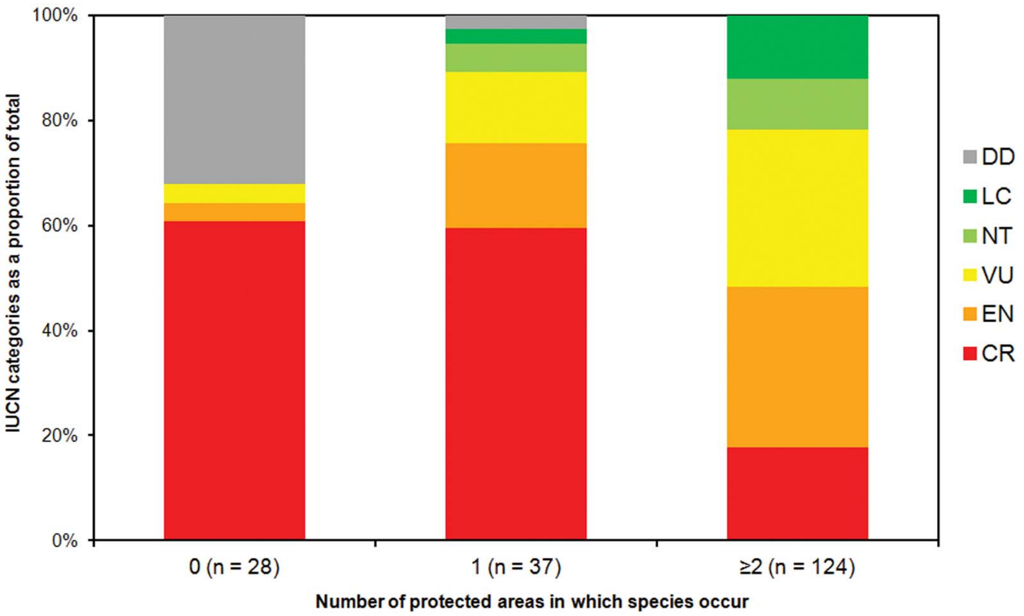

Figure 4. IUCN conservation status of palm species summarised by occurrence in protected areas (SAPM network). Of the 192 assessed species [44], 28 are not recorded from any protected area (coded as 0 in the figure), 37 species are recorded only from one protected area (coded as 1) and 124 species occur in two or more protected areas (coded as ≥2). Three data deficient species are not included as their distributions are unknown (Dypsis lucens, D. plumosa and D. thouarsiana).
doi:10.1371/journal.pone.0103684.g004

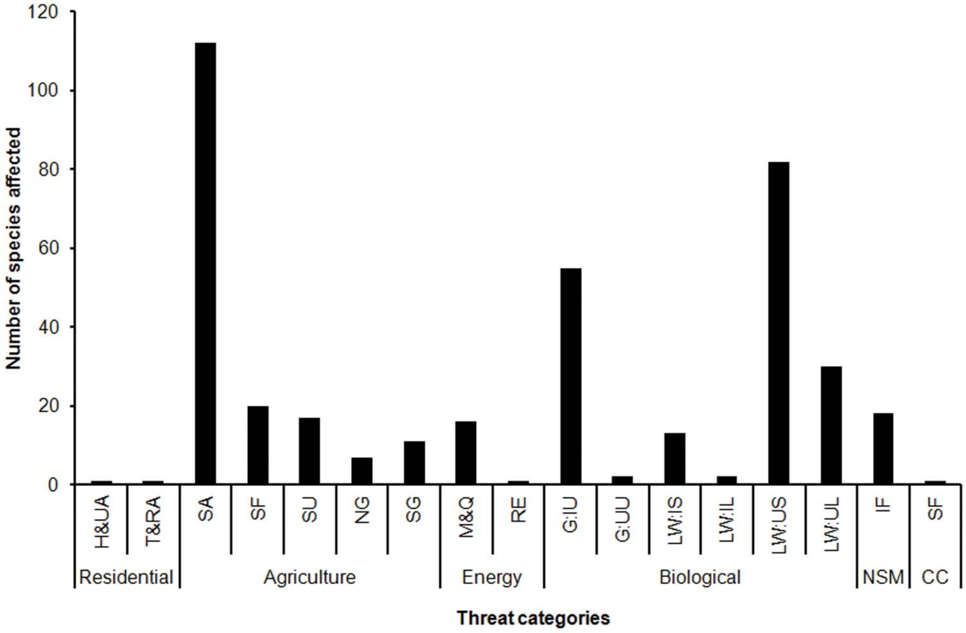

Figure 5. Major threats affecting endemic palm species in Madagascar. Bar heights reflect number of species affected by each threat, as indicated in the 2012 IUCN conservation assessment [44]. Threat categories follow the Threats Classification Scheme version 3.2 [51], using the top two levels of the hierarchy. Abbreviations: Residential & commercial development (Residential): Housing & urban areas (H&UA), Tourism & recreation areas (T&RA); Agriculture & aquaculture (Agriculture): Shifting agriculture (SA), Small-holder farming (SF), Scale unknown/unrecorded (SU), Nomadic grazing (NG), Small-holder grazing, ranching or farming (SG); Energy production & mining (Energy): Mining & quarrying (M&Q), Renewable energy (RE); Biological resource use (Biological): Gathering terrestrial plants, Intentional use (species being assessed is the target) (G:IU), Gathering terrestrial plants, unintentional use (G:UU), Logging & wood harvesting for subsistence, Intentional use: subsistence/small scale (species being assessed is the target) (LW: IS), Logging & wood harvesting, Intentional use: large scale (species being assessed is the target) (LW: IL); Logging & wood harvesting, Unintentional effects: subsistence/small scale (species being assessed is not the target) (LW: US), Logging & wood harvesting, Unintentional effects: large scale (species being assessed is not the target) (LW: UL); Natural system modifications (NSM): Increase in fire frequency (IF); Climate change & severe weather (Climate): Storm & flooding (SF).
doi:10.1371/journal.pone.0103684.g005

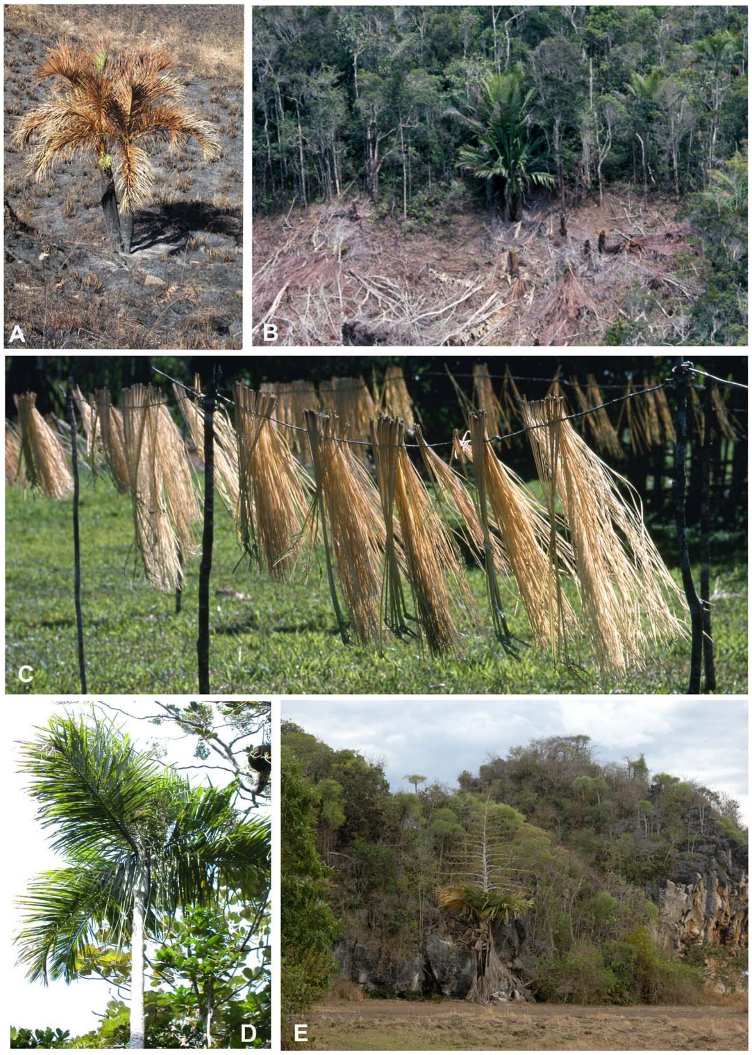

Figure 6. Example of palm species under threat in Madagascar. (a) Anthropogenic fires in grasslands, causing decline and destruction of palm populations, such as *Dypsis decipiens* (VU), Itremo. (b) Forest clearance for slash and burn cultivation by smallholder farmers, causing habitat loss for many species, such as *Masoala kona* (EN), Ifanadiana. (c) Gathering of young leaves of *Ravenea lakatra* (VU) for production of woven hats and basketry, Masoala. (d) Destructive harvest of palm heart threatens many species such as *Dypsis saintelucei* (EN), Sainte Luce. (e) Remnant populations of species such as *Tahina spectabilis* (CR), Analalava, near Mahajanga in vegetation remnants isolated within anthropogenic landscapes, at risk from fire, grazing and other human pressures. Image credits: (a) M Rakotoarinivo, (b) WJ Baker, (c, d & e) J Dransfield.
doi:10.1371/journal.pone.0103684.g006

Manompana), further emphasising the importance of the expanded SAPM network. These protected areas vary widely in extent, but all are located in the humid forested east. Nevertheless, protected areas do not guarantee low extinction risk as the majority of species that occur in protected areas are still assessed as threatened (Fig. 4), indicating that threatening processes persist in these areas.

Threats

The major threatening processes for palms in Madagascar are agriculture and biological resource use with 167 and 184 species affected by these threats respectively. More specifically the threats to palm habitats from agriculture relate to annual and perennial non-timber crop production i.e. crops planted for food, fodder, fibre, fuel or other uses, with 'shifting agriculture' listed as the scale of farming affecting the highest number of species (112) (Figs. 5 and 6; threat wordings according to IUCN Threats Classification Scheme (version 3.2) [51]). The threat from biological resource use is related to the gathering of terrestrial plants (55 species, e.g. for palm heart consumption) and logging and wood harvesting (127 species). More specifically the highest scoring threat is from logging and wood harvesting for subsistence on a large scale where the species of palm is actually not intended target, but is threatened due to collateral damage (112 species) i.e. the palms are subject to collateral damage. Other less prevalent threats relate to mining, livestock farming, fires, housing and urban development.

Discussion

As of 2013, 684 native plant species from Madagascar (out of an estimated total of ca. 13,000 [55]) were completely assessed under the IUCN categories and criteria [31] and displayed on the

website of the Red List [44]. With 192 species and representing nearly 30% of the Madagascar plant species on the Red List, our study of palms is the largest and most complete IUCN conservation assessment of any plant family on the island. Recently, the IUCN Madagascar Plant Specialist Group (Groupe des Spécialistes des Plantes Malgaches, GSPM) [55] assessed ca. 3,000 plant species from 74 families and 285 genera. In addition, full assessments of smaller taxonomic groups have been completed: Pandanaceae (91 species [25]), Sarcolaenaceae (68 species) and Sphaerosepalaceae (20 species) [56], tribe Coleeae of Bignoniaceae (67 species [57]) and the genus *Delonix* (Fabaceae, 11 species; [58]). Unfortunately, none of these groups of assessments has been formally published on the IUCN Red List yet.

Our finding that as many as 83% of palm species are threatened (best estimate) indicates that the Arecaceae is among the organismal groups facing the highest risk of extinction in Madagascar. Moreover, the proportion of threatened palms in Madagascar is almost four times greater than for plants in general, estimated as 21.5% worldwide [59], and is higher than the estimate for Madagascar's flora as a whole (54%, [60]). At family level, the percentage of threatened species is variable, 49% of all endemic legumes [57], 65% for Sphaerosepalaceae and 75% for Sarcolaenaceae [56], and 81.3% for Pandanaceae [25]. It is notable that the proportions of threatened species in the Arecaceae and Pandanaceae are similar, given that they share functional similarities as woody, often arborescent monocotyledonous plants that are most species rich in humid forests. For major groups of animals in Madagascar, the proportion of threatened species varies widely, for example, 25% for amphibians [61], 37% for reptiles [60] and 94% for lemurs [62].

Comparisons between the 1995 and 2012 assessments of Madagascar palms [10] indicates that downlisting (a movement to a category of lower threat) is more frequent than uplisting, though 21 of 32 downlisted species still fall into threatened categories. These changes to a lower category come from improved knowledge of species distribution, population size, and taxonomy rather than any genuine decline of extinction risk in the wild. In contrast, almost all of our recently discovered species (species not known to science in 1995) are threatened as they typically have small range sizes and are at risk of habitat loss and direct or indirect threats from human pressure [63]. The role of the taxonomist in conservation assessment cannot be overstated as collections-based research and knowledge both in the field and in the herbarium or museum is essential for confirming the identity of species and the distribution of their wild populations. Conservation assessment in the absence of robust taxonomy will result in inaccurate ratings and a tendency to categorise species as DD [57,64]. In our case, intensive taxonomic research and field surveys in Madagascar fundamentally underpin our conservation assessments and have led to the rediscovery of species previously thought to be extinct, as well as the discovery of new populations of threatened species and species new to science.

Our analysis of threats facing palms suggests that the dependency of rural people on forested lands for shifting cultivation and their continued unsustainable exploitation of wild forest products such as palms are key drivers of palm extinction risk. This applies even in remote areas where human population density is low [35]. Our analysis also reveals a novel and insidious threat to palms through the logging or harvesting of other plants at a subsistence level that causes collateral damage to palms. Unless the economic circumstances of rural communities change radically, forest resources such as palms will continue to be exploited unsustainably for basic subsistence needs. Economic factors are a primary concern for the conservation of Madagascar palms, as they are for so many other organisms globally.

Time-delayed biodiversity loss [65] is an important consideration for Madagascar palms as many species persist locally as seedlings or juvenile plants after mature trees have been cleared with the forest. Species in decline may survive for a long time before they become extinct if a threshold in the habitat quality is maintained [66]. Without adequate protection and management, these sites are likely to be lost in the future as disturbance and fragmentation provide suitable habitat for invasive secondary species [12], which have negative impacts on native species by depressing the growth rate at various stages of the life cycle [67].

The high degree of extinction risk faced by Madagascar palms calls into question the effectiveness of previous conservation actions on the island. In a period when human population density and pressure on biodiversity are increasing, the long-term success of protected areas is at the heart of potential solutions for palm conservation. By covering 70% of the remaining humid forest in Madagascar [68], the SAPM network is expected to be considerably more effective for species protection in Madagascar compared with the previous, more limited MNP network, as the new set of reserves has been selected to include narrow range taxa [69]. Our analysis demonstrates that SAPM protects threatened palm populations much more effectively than the MNP network. Nevertheless, the SAPM network has limitations. To date, only Makira has been accorded definitive protected area status, whereas the remainder are not yet formally designated [23]. Consequently, critical protection of the forest and its biodiversity is lacking in the majority of the SAPM network.

Moreover, SAPM does not provide complete protection for Madagascar palms as several priority sites (Table 4), typically forest fragments far from protected areas, are not included. Small areas of intact habitat need to be taken into account as they often contain remnant populations of rare and endemic species [70] that are highly susceptible to environmental stochasticity and local extinction [71]. For example, a monotypic genus of massive fan palm, *Tahina spectabilis*, discovered only in 2006 [72], persists in a 160 × 50 m patch of forested tsingy (karst limestone) surrounded by anthropogenic grassland near Mahajanga. The protection of this forest is an urgent priority to conserve this isolated, endemic lineage. Some small fragments are included in the SAPM network, such as a ca. 2 km^2 tract of degraded coastal plain forest at Analalava (near Foulpointe), north of Toamasina, which is an outstanding palm hotspot containing 25 palm species, including one local endemic and three species known from only one other site each. This small fragment is managed locally by the Missouri Botanical Garden staff who promote the site for ecotourism [73].

Madagascar palms face exceptional levels of extinction risk by both national and global standards. The conservation of keystone species such as palms [9] is of particular importance due to the potential consequences of their extinction to other species. Humans are among the organisms that rely substantially on ecosystem services provided by palms [10,15,74,75]. The engagement of local communities in conservation initiatives will be critical to their success. Given the intensifying pressure from growing human populations, compounded by projected impacts of climate change on species extinction [76], there is now an urgent need for prioritised action for Madagascar palms. The rigorous IUCN conservation assessment described here provides an essential foundation for such a process.

Acknowledgments

We thank Craig Hilton-Taylor and Henk Beentje for their support and advice during the Red Listing process, and Charlotte Rajeriarison and

Stuart Cable for supervision. We acknowledge the cooperation of the IUCN SSC Madagascar Plant Specialist Group, particularly Sylvie Andriambololonera. Martin Callmander and Pete Lowry provided many useful comments on an earlier draft of the manuscript. We are grateful to the many authorities, institutions and individuals who have supported and facilitated our fieldwork.

Author Contributions

Conceived and designed the experiments: MR JD SPB JM WJB. Performed the experiments: MR JD SPB JM WJB. Analyzed the data: MR JD SPB JM WJB. Contributed reagents/materials/analysis tools: MR JD SPB JM WJB. Wrote the paper: MR JD SPB JM WJB.

References

1. Myers N, Mittermeier RA, Mittermeier CG, da Fonseca GA, Kent J (2000) Biodiversity hotspots for conservation priorities. Nature 403: 853–858. doi:10.1038/35002501.
2. Goodman SM, Benstead JP (2005) Updated estimates of biotic diversity and endemism for Madagascar. Oryx 39: 73–77 doi: 10.1017/S0030605305000128.
3. Harper GJ, Steininger MK, Tucker CJ, Hawkins F (2007) Fifty years of deforestation and forest fragmentation in Madagascar. Environ Conserv 34: 325–333. doi: 10.1017/S0376892907004262.
4. Allnutt TF, Asner GP, Golden CD, Powell GV (2013) Mapping recent deforestation and forest disturbance in northeastern Madagascar. Trop Conserv Sci 6: 1–15.
5. Govaerts R, Dransfield J, Zona SF, Hodel DR, Henderson A (2012) World Checklist of Arecaceae. Facilitated by the Royal Botanic Gardens, Kew. Available: http://apps.kew.org/wcsp/. Accessed 12 June 2013.
6. Rakotoarinivo M, Blach-Overgaard A, Baker WJ, Dransfield J, Moat J, et al. (2013) Palaeo-precipitation determines palm diversity across Madagascar - a tropical biodiversity hotspot. Proc R Soc Lond B Biol Sci 280: 20123048. doi:10.1098/rspb.2012.3048.
7. Kissling WD, Eiserhardt WL, Baker WJ, Borchsenius F, Couvreur TLP, et al. (2012) Cenozoic imprints on the phylogenetic structure of palm species assemblages worldwide. Proc Natl Acad Sci USA 109: 7379–7384. doi:10.1073/pnas.1120467109.
8. Couvreur T, Forest F, Baker W (2011) Origin and global diversification patterns of tropical rain forests: inferences from a complete genus-level phylogeny of palms. BMC Biol 9: 44. doi:10.1186/1741-7007-9-44.
9. Couvreur T, Baker W (2013) Tropical rain forest evolution: palms as a model group. BMC Biol 11: 48. doi:10.1186/1741–7007–11–48.
10. Dransfield J, Beentje H (1995) The palms of Madagascar. The Royal Botanic Gardens, Kew and The International Palm Society. 475 p.
11. Fleischmann K, Edwards PJ, Ramseier D, Kollmann J (2005) Stand structure, species diversity and regeneration of an endemic palm forest on the Seychelles. Afr J Ecol 43: 291–301. doi: 10.1111/j.1365–2028.2005.00567.x.
12. Scariot A (1999) Forest fragmentation effects on palm diversity in central Amazonia. J Ecol 87: 66–76. doi: 10.1046/j.1365–2745.1999.00332.x.
13. Moat J, Smith P (2007) Atlas of the Vegetation of Madagascar. Kew Publishing. 124p.
14. Bennett BC (2011) Twenty-five economically important plant families. Encyclopedia of Life Support. Available: http://www.eolss.net/sample-chapters/c09/e6-118-03.pdf. Accessed 15May 2013.
15. Dransfield J, Uhl NW, Asmussen CB, Baker WJ, Harley MM, et al. (2008) Genera Palmarum – the evolution and classification of palms. Kew Publishing. 744 p.
16. Dransfield J (1999) Madagascar as a source of new palm introductions. Acta Hortic 486: 21–32.
17. Isaac NJ, Turvey ST, Collen B, Waterman C, Baillie JE (2007) Mammals on the EDGE: Conservation priorities based on threat and phylogeny. PLoS ONE 2: e296. doi:10.1371/journal.pone.0000296.
18. Richard D, Evans D (2006) The need for plant taxonomy in setting priorities for designated areas and conservation management plans: a European perspective. In: Leadlay E, Jury S, editors. Taxonomy and Plant conservation. Cambridge University Press. pp. 163–176.
19. Bruner AG, Gullison RE, Rice RE, da Fonseca GA (2001) Effectiveness of parks in protecting tropical biodiversity. Science 291: 125–128. doi: 10.1126/science.291.5501.125.
20. Borrini-feyerabend G, Dudley N(2005) Les Aires Protégées à Madagascar : bâtir le système à partir de la base. World Commission on Protected Areas & International Union for Conservation of Nature. 51 p.
21. Norris S (2006) Madagascar defiant. BioScience 56: 960–960. doi:10.1641/0006–3568(2006)56[960:MD]2.0.CO;2.
22. Mittermeier RA, Hawkins F, Rajaobelina S, Langrand O (2005) Wilderness conservation in a biodiversity hotspot. International Journal of Wilderness 11: 42–45.
23. Système des Aires Protégées de Madagascar [SAPM] (2011) Atlas numerique du SAPM. Available : http://atlas.rebioma.net/index.php?option = com_wrapper&Itemid = 39. Accessed 3 June 2013.
24. Nicoll ME (2003) Forests outside protected areas In: Goodman SM, Benstead JP, editors. The Natural History of Madagascar. University of Chicago Press. pp. 1432–1437.
25. Callmander MW, Schatz GE, Lowry II PP, Laivao MO, Raharimampionona J, et al. (2007) Identification of priority areas for plant conservation in Madagascar using Red List criteria: rare and threatened Pandanaceae indicate sites in need of protection. Oryx 41: 168–176. doi: 10.1017/S0030605307001731.
26. Rodrigues ASL, Pilgrim JD, Lamoreux JF, Hoffmann M, Brooks TM (2006) The value of the IUCN Red List for conservation. Trends Ecol Evol 21: 71–76. doi:10.1016/j.tree.2005.10.010.
27. Mace G, Collar N, Gaston K, Hilton-Taylor C, Akçakaya R, et al. (2008) Quantification of extinction risk: IUCN's system for classifying threatened species. Conserv Biol 22: 1424–1442. doi: 10.1111/j.1523–1739.2008.01044.x.
28. Vié JC, Hilton-Taylor C, Pollock C, Ragle J, Smart J, et al. (2008) The IUCN Red List: a key conservation tool. IUCN, Switzerland. 13 p.
29. International Union for Conservation of Nature [IUCN] (1994) IUCN Red List Categories and Criteria version 2.3. Available: http://www.iucnredlist.org/technical-documents/categories-and-criteria/1994-categories-criteria. Accessed 29 May 2013.
30. International Union for Conservation of Nature [IUCN] (1998) 1997 IUCN Red List of Threatened Plants. World Commission on Protected Areas [WCMC] & International Union for Conservation of Nature [IUCN]. 862 p.
31. International Union for Conservation of Nature [IUCN] (2001) IUCN Red List Categories and Criteria: Version 3.1. IUCN, Gland, Switzerland and Cambridge, UK. 30 p.
32. Dransfield J, Beentje H, Britt A, Ranarivelo T, Razafitsalama J (2006) Field guide to the palms of Madagascar. Royal Botanic Gardens, Kew. 172 p.
33. Rakotoarinivo M, Trudgen MS, Baker WJ (2009) The palms of Makira protected areas. Palms 53: 125–146.
34. Rakotoarinivo M, Dransfield J (2010) New species of Dypsis and Ravenea (Arecaceae) from Madagascar. Kew Bull 65: 279–303.
35. Foibe Taosarintanin'i Madagasikara [FTM] (2006) Madagasikara et ses 22 régions. FTM. 24 p.
36. Battistini R (1996) Paléogéographie et variété des milieux naturels à Madagascar et dans les îles voisines : quelques données de base pour l'étude biogéographique de la région malgache. In: Lourenço WR, editor. Biogéographie de Madagascar, ORSTOM, Paris. pp. 1–17.
37. Jury MR (2003) The Climate of Madagascar. In: Goodman SM, Benstead JP, editors. The Natural History of Madagascar. University of Chicago Press. pp. 75–87.
38. WorldBank (2013) Population density. Available: http://data.worldbank.org/country/madagascar. Accessed 31 May 2013.
39. Allnutt TF, Ferrier S, Manion G, Powell GVN, Ricketts TH, et al. (2008) A method for quantifying biodiversity loss and its application to a 50-year record of deforestation across Madagascar. Cons Lett 1: 173–181. doi: 10.1111/j.1755–263X.2008.00027.x.
40. Thiers B (2012) Index Herbariorum, a global directory of public herbaria and associated staff. New York Botanical Garden's virtual herbarium. Available: http://sweetgum.nybg.org/ih/. Accessed 26 April 2013.
41. Schatz G, Lescot M (2003) Gazetteer to Malagasy Botanical Collecting Localities. Missouri Botanical Garden Available: http://www.mobot.org/MOBOT/Research/madagascar/gazetteer/. Accessed 15 July 2012.
42. Google (2010) Google Earth (version 5.2.1.1588). Available: http://www.google.com/earth/explore/products/. Accessed 03 November 2011.
43. International Union for Conservation of Nature [IUCN] Standards and Petitions Subcommittee (2011) Guidelines for Using the IUCN Red List Categories and Criteria. Version 9.0. Prepared by the Standards and Petitions Subcommittee. Available : http://www.iucnredlist.org/documents/RedListGuidelines.pdf. Accessed: 06 July 2013.
44. IUCN (2012) IUCN Red List of Threatened Species. Version 2013.1. Available: http://www.iucnredlist.org Accessed 10 October 2013.
45. Gaston KJ, Fuller RA (2009) The sizes of species' geographic ranges. J Appl Ecol 46: 1–9. doi: 10.1111/j.1365–2664.2008.01596.x.
46. Willis F, Moat J, Paton A (2003) Defining a role for herbarium data in Red List assessments : a case study of *Plectranthus* from eastern and southern tropical Africa. Biodivers Conserv 12: 1537–1552. doi: 10.1023/A:1023679329093.
47. Bachman S, Moat J, Hill AW, de la Torre J, Scott B (2011) Supporting Red List threat assessments with GeoCAT: geospatial conservation assessment tool. Zookeys 150: 117–126. doi: 10.3897/zookeys.150.2109.
48. Moat J (2007) Conservation assessment tools extension for ArcView 3.x, version 1.2. GIS Unit, Royal Botanic Gardens, Kew. Available: http://www.rbgkew.org.uk/gis/cats. Accessed 12 November 2012.
49. Humbert H, Cours-Darne G (1965) Notice de la carte de Madagascar : carte internationale du tapis végétal et des conditions écologiques à 1:1.000.000. Institut Français de Pondichéry. 162 p.
50. Association Nationale pour la Gestion des Aires Protégées [ANGAP] (2001) Madagascar Protected Area System Management Plan. Mye. 112 p.
51. International Union for Conservation of Nature [IUCN] (2012) Threats Classification Scheme (Version 3.2). Available: http://www.iucnredlist.org/technical-documents/classification-schemes/ threats-classification-scheme. Accessed 10 May 2013.

52. Hoffmann M, Hilton-Taylor C, Angulo A, Böhm M, Brooks TM, et al. (2010) The Impact of Conservation on the Status of the World's Vertebrates. Science 330: 1503–1509. doi: 10.1126/science.1194442.
53. Butchart SH, Akcakaya HR, Kennedy E, Hilton-Taylor C (2006) Biodiversity indicators based on trends in conservation status: strengths of the IUCN Red List index. Conserv Biol 20: 579–581. doi: 10.1111/j.1523-1739.2006.00410.x.
54. Butchart SH, Resit Akçakaya H, Chanson J, Baillie JE, Collen B, et al. (2007) Improvements to the Red List Index. PLoS ONE 2: e140. doi:10.1371/journal.pone.0000140.
55. Groupe des Spécialistes des Plantes Malgaches [GSPM] (2011) Liste rouge des plantes vasculaires endemiques de Madagascar. Sud Expert Plantes, Antananarivo. 188 p.
56. Ramananjanahary RH, Frasier CL, Lowry II PP, Rajaonary FA, Schatz GE (2010) Madagascar's endemic plant families, species Guide. Missouri Botanical Garden, Madagascar. 150 p.
57. Good TC, Zjhra ML, Kremen C (2006) Addressing data deficiency in classifying extinction risk: a case study of a radiation of Bignoniaceae from Madagascar. Conserv Biol 20: 1099–1110. doi: 10.1111/j.1523–1739.2006.00473.x.
58. Rivers MC, Bachman SP, Meagher TR, Nic Lughadha E, Brummitt NA (2010) Subpopulations, locations and fragmentation: applying IUCN red list criteria to herbarium specimen data. Biodivers Conserv 19: 2071–2085. doi: 10.1007/s10531-010-9826-9.
59. Brummitt N, Bachman S (2010) Plants under pressure a global assessment. The first report of the IUCN Sampled Red List Index for plants. Royal Botanic Gardens, Kew, UK. Available : http://www.kew.org/ucm/groups/public/documents/document/kppcont_082104.pdf. Accessed 14 September 2012.
60. International Union for Conservation of Nature (2013) The IUCN Red List of threatened species - Summary statistics. Available: http://www.iucnredlist.org/documents/summarystatistics/2013_1_RL_Stats_Table_5.pdf. Accessed 25 October 2013.
61. Andreone F, Cadle JE, Cox N, Glaw F, Nussbaum RA, et al. (2005) Species review of Amphibians extinction risks in Madagascar: conclusions from the global Amphibians assessment. Conserv Biol 19: 1790–1802. doi: 10.1111/j.1523–1739.2005.00249.x.
62. Davies N, Schwitzer C (2013) Lemur conservation status review: an overview of the Lemur Red-Listing results 2012. In: Schwitzer C, Mittermeier RA, Davies N, Johnson S, Ratsimbazafy J, et al., editors. Lemurs of Madagascar: A Strategy for their Conservation 2013–2016. IUCN SSC Primate Specialist Group, Bristol Conservation and Science Foundation, and Conservation International pp. 13–33.
63. Joppa LN, Roberts DL, Myers N, Pimm SL (2011) Biodoversity hotspots house most undiscovered plant species. P Natl Acad Sci USA 108: 13171–13176. doi:10.1073/pnas.1109389108.
64. Callmander MW, Schatz GE, Lowry II PP (2005) UICN Red List assessment and the Global Strategy for plant conservation : taxonomists must act now. Taxon 54: 1047–1050. doi: 10.2307/25065491.
65. Krauss J, Bommarco R, Guardiola M, Heikkinen RK, Helm A, et al. (2010) Habitat fragmentation causes immediate and time-delayed biodiversity loss at different trophic levels. Ecol Lett 13: 597–605. doi: 10.1111/j.1461–0248.2010.01457.x.
66. Fattorini S, Borges PAV (2012) Species-area relationships underestimate extinction rates. Acta Oecol 40: 27–30. doi:10.1038/nature09985.
67. Rojas-Sandoval J, Meléndez-Ackerman E (2012) Effects of an invasive grass on the demography of the Caribbean cactus *Harrisia portoricensis*: Implications for cacti conservation. Acta Oecol 41: 30–38. doi: 10.1016/j.actao.2012.04.004.
68. Hannah L, Dave R, Lowry II PP, Andelman S, Andrianarisata M, et al. (2008) Climate change adaptation for conservation in Madagascar. Biol Letters 4: 590–594. doi:10.1098/rsbl.2008.0270.
69. Kremen C, Cameron A, Moilanen A, Philips SJ, Thomas CD, et al. (2008) Aligning conservation priorities across taxa in Madagascar with high resolution planning tools. Nature 230: 222–226. doi: 10.1126/science.1155193.
70. Marcot BG, Raphael MG, Schumaker NH, Galleher B (2013) How big and how close? habitat patch size and spacing to conserve a threatened species. Nat Resour Model 26: 194–214. doi: 10.1111/J.1939–7445.2012.00134.X.
71. Hobbs RJ (2007) Setting effective and realistic restoration goals: key directions for research. Restor Ecol 15 : 354–357. doi: 10.1111/j.1526-100X.2007.00225.x.
72. Dransfield J, Rakotoarinivo M, Baker WJ, Bayton RP, Fisher JB, et al. (2008). A new Coryphoid palm genus from Madagascar. Bot J Linn Soc 156: 79–91. doi: 10.1111/j.1095-8339.2007.00742.x.
73. Rakotoarinivo M, Razafitsalama JL, Baker W, Dransfield J (2010) Analalava – a palm conservation hotspot in eastern Nadagascar. Palms 54: 141–151.
74. Byg A, Balslev H (2001) Diversity and use of palms in Zahamena, eastern Madagascar. Biodivers Conserv 10: 951–970. doi:10.1023/A:1016640713643.
75. Byg A, Balslev H (2003) Palm heart extraction in Zahamena, Eastern Madagascar. Palms 47: 37–44.
76. Thomas CD, Cameron A, Green RE, Bakkenes M, Beaumont LJ, et al. (2004) Extinction risk from climate change. Nature 427: 145–148. doi:10.1038/nature02121.
